# Low-Temperature Photothermal Therapy: Strategies and Applications

**DOI:** 10.34133/2021/9816594

**Published:** 2021-05-07

**Authors:** Xiulin Yi, Qiu-Yi Duan, Fu-Gen Wu

**Affiliations:** State Key Laboratory of Bioelectronics, School of Biological Science and Medical Engineering, Southeast University, 2 Sipailou Road, Nanjing 210096, China

## Abstract

Although photothermal therapy (PTT) with the assistance of nanotechnology has been considered as an indispensable strategy in the biomedical field, it still encounters some severe problems that need to be solved. Excessive heat can induce treated cells to develop thermal resistance, and thus, the efficacy of PTT may be dramatically decreased. In the meantime, the uncontrollable diffusion of heat can pose a threat to the surrounding healthy tissues. Recently, low-temperature PTT (also known as mild PTT or mild-temperature PTT) has demonstrated its remarkable capacity of conquering these obstacles and has shown excellent performance in bacterial elimination, wound healing, and cancer treatments. Herein, we summarize the recently proposed strategies for achieving low-temperature PTT based on nanomaterials and introduce the synthesis, characteristics, and applications of these nanoplatforms. Additionally, the combination of PTT and other therapeutic modalities for defeating cancers and the synergistic cancer therapeutic effect of the combined treatments are discussed. Finally, the current limitations and future directions are proposed for inspiring more researchers to make contributions to promoting low-temperature PTT toward more successful preclinical and clinical disease treatments.

## 1. Introduction

In the past few decades, photothermal therapy (PTT) which usually utilizes photothermal agents (PTAs) capable of generating abundant heat under light irradiation has been frequently applied to kill/inactivate tumors [1] or bacteria [2], and is thus acknowledged as one of the primary treatment strategies in clinical and preclinical phases. With the rapid development of nanotechnology, various nanomaterials have been constructed for a broad range of biomedical applications and have exhibited excellent performance in treating a variety of diseases, including bacterial infection [3], cancer [4], neural diseases [5], and cardiovascular diseases [6]. Compared to traditional thermal therapy methods, the nanomaterial-mediated hyperthermia can realize bacterium- or tumor-specific location by designing passive and/or active targeting nanoplatforms and achieve on-demand treatments via controlling the external energy sources like light. Under the external energy sources, the nanomaterials located at the targeted tissues can produce heat and the inside-out heating direction remarkably decreases undesirable damage to normal tissues, and moreover, the nanomaterial-mediated hyperthermia therapy holds great promise for spatiotemporally controllable disease treatments [1, 2, 7]. Additionally, especially for cancer treatments, PTT based on nanotechnology has been combined with other therapeutic modalities including chemotherapy, radiotherapy (RT), photodynamic therapy (PDT), gene therapy, immunotherapy, and chemodynamic therapy (CDT) to achieve synergistic treatments [8–10], which has been considered as a robust strategy for improving therapeutic efficacies. In the past decades, researchers have found a large number of nanomaterials possessing photothermal conversion capacity, which can be categorized into two groups—inorganic PTAs and organic PTAs. The inorganic PTAs include metal or metal-containing nanomaterials including gold nanostructures [11, 12], palladium-based nanostructures [13–15], iron or copper-containing nanoparticles (NPs) [16–19], transition metal chalcogenides [20], and quantum dots [21, 22]. On the other hand, organic materials [23] including near-infrared (NIR) dyes represented by cyanine dyes [24–32], conjugated polymers (e.g., polydopamine (PDA), polyaniline, polypyrrole, and poly(3,4-ethylenedioxythiophene)) [33–36], and some carbon-based nanomaterials (most of them are organic materials; as represented by graphite-related nanostructures) [37–41] have also been utilized for PTT.

There are several properties that dictate the satisfactory PTT efficacy of PTAs: (1) strong absorbance in the NIR region and superb photothermal conversion capacity, (2) acceptable biocompatibility and biosafety, and (3) the accessibility of surface modification for realizing quality improvement and multifunctionality. For (3), in terms of conquering bacterial infections, factors like the balance between hydrophobicity and cationic charges of nanomaterials should be considered; as for cancer treatments, factors including prolonged circulation time, low retention in liver and kidney, and pronounced tumor-accumulating/targeting ability for precise therapy need to be emphasized. Compared with other conventional therapeutic approaches, PTT possesses the typical advantages in terms of noninvasiveness, negligible toxicity to normal cells in the dark, and spatiotemporally controlled administration. Besides, through the employment of NIR light irradiation, much deeper tissue penetration for treating interior or deep-seated tumors, bacterial infections, or wounds can be achieved.

However, excessive hyperthermia poses an unavoidable threat to surrounding healthy tissues and may induce undesirable inflammation because of the difficulty in blocking heat diffusion. Moreover, in cancer treatments, there are some undesirable biological effects induced by high-temperature thermal ablation. On the one hand, since PTT that takes effect at a temperature above 50°C compels cells to death mainly through necrosis [7], which is supposed to cause the release of cellular fragments and intracellular biomolecules, violent local inflammation may appear resulting in the further damage to normal tissues and the increase in tumor metastasis [42]. On the other hand, it is considered that overheating at such high temperatures has the risk of impairing the immune antigens capable of evoking antitumor immunity and immune cells exceuting immune responses in the tumor microenvironment (TME), thus preventing immune system from conquering cancers [43, 44]. Further, to reach a temperature high enough to thoroughly kill cancer cells or bacteria requires high-quality NIR lasers and/or high-performance PTAs, which may increase the cost/consumption and then restrain the application in clinic.

For surmounting these bottlenecks, low-temperature PTT (also termed mild-temperature PTT or mild PTT) which preferentially eliminates bacteria or tumors and promotes wound healing under low temperatures with inconsiderable damage to normal tissues and inflammation has been proposed. A large number of studies have elucidated that low-temperature PTT can achieve fantastic therapeutic performance to enable practical biomedical applications. Although there are different temperature thresholds of low-temperature PTT, it is widely recognized that most of the mild hyperthermia treatments are conducted at temperatures below 48°C [7, 45]. Meanwhile, low-temperature PTT can be achieved under mild NIR laser irradiation with a laser density not higher than the permitted value 0.329 W/cm^2^ (for 808 nm) for skin exposure [46]. Although it has been widely considered that the efficacy of mild hyperthermia can be dramatically compromised due to the elevated expression of heat shock proteins (HSPs) able to repair the heat injury and protect cells from apoptosis through multiple stress-related pathways, which leads to tumor thermal resistance [45, 47], there have been various strategies proven to be capable of solving this problem. Importantly, a large number of studies have demonstrated that mild heat can render the cancer cells and bacteria more vulnerable to other treatment modalities, thus elevating the effectiveness of other treatments [44, 45]. For example, hyperthermic perfusion chemotherapy (including hyperthermic isolated limb perfusion and hyperthermic intraperitoneal chemotherapy) systems have been utilized for cancer treatments in clinic for many years and exhibited satisfactory therapeutic effects [48, 49]. Furthermore, similar to conventional PTT, the rapid development of nanotechnology enables the use of various nanomaterials for achieving low-temperature PTT.

**Figure 1:**
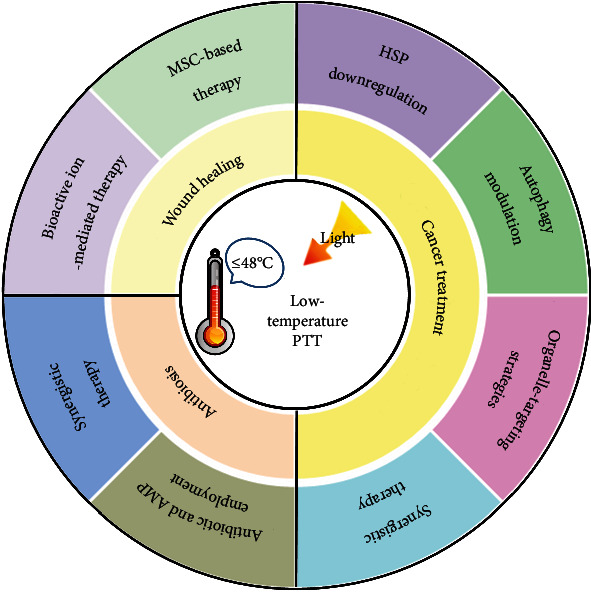
Scheme illustrating the use of low-temperature PTT for antibiosis, wound healing, and cancer treatment via various strategies.

In this review, we comprehensively summarize and comment on the recent advances regarding the development of strategies for realizing low-temperature PTT and the utilization of low-temperature PTT for combating tumors and bacterial infections and facilitating wound healing (Figure 1). It is believed that the summarization of the low-temperature PTT strategies will inspire more researchers to adopt such an efficacious treatment modality to treat a variety of diseases (beyond bacterial infections, wounds, and cancers). Regarding the applications of low-temperature PTT, we will divide this part into three sections: (1) low-temperature PTT for treating bacterial infections, (2) low-temperature PTT for promoting wound healing, and (3) low-temperature PTT for cancer treatments. For the first and second parts, we will introduce the preparation and composition of the reported platforms and discuss the effectiveness of mild PTT in antibiosis and wound healing applications. For the third section, we will pay specific attention to the proposed strategies for realizing effective mild PTT and the combined uses of low-temperature PTT and other cancer therapeutic modalities for achieving enhanced anticancer outcomes. Finally, we will list the current challenges faced in this field and propose some future research directions aiming at improving the existing strategies and developing new strategies for realizing low-temperature PTT.

## 2. Applications of Low-Temperature PTT

### 2.1. Low-Temperature PTT for Treating Bacterial Infections

There are various kinds of pathogenic bacteria posing severe threats to human health, and the fight against the infections caused by these bacteria has become one of the most important biomedical directions for many decades [50]. Although the development of antibiotics has relieved the unfavorable condition to a considerable extent, we have not conquered bacterial infections fundamentally, especially in the situation where the bacteria have the staggering capability of mutation to adapt to extremely harsh environments resulting in the transformation to antibiotic-resistant bacteria. Additionally, many bacteria live in a sticky and multicellular community called biofilm which consists of plentiful biochemical molecules making an indispensable contribution to bacterial growth and reproduction [51]. The biofilms serve as a natural obstacle to prevent the invasion of life-threatening substances including antibiotics and some designed antibacterial nanomaterials. Accordingly, developing effective and practical methods able to surmount bacterial infections thoroughly is a challenging and urgent task.

PTT associated with nanotechnology has been well employed for antibacterial applications in recent decades, due to its broad-spectrum bacterial killing activity, avoidance of drug resistance, and satisfactory controllability. It is widely recognized that the mechanism of employing hyperthermia for killing microorganisms is destroying diverse life-fundamental molecules or structures, including protein denaturation, lipid evaporation, and cell membrane breakage [2, 52]. To date, many kinds of PTAs, such as carbon-based nanomaterials [53, 54], noble metal nanomaterials [55, 56], other metal-containing nanocomposites [57], and conjugated polymers [35, 36], have been used for antimicrobial applications and have established their unique advantages. Moreover, owing to the uneven heat distribution in the bacterial biofilms, it is difficult to completely eradicate the bacteria within the biofilms via PTT alone. In recent years, as shown in Table 1, an increasing number of related studies have demonstrated that the combination of low-temperature PTT and other therapeutic modalities including PDT and gas therapy or the combination of PTT and antimicrobial peptides (AMPs) or antibiotics has a remarkable performance in eliminating bacteria [58–61].

**Figure 2:**
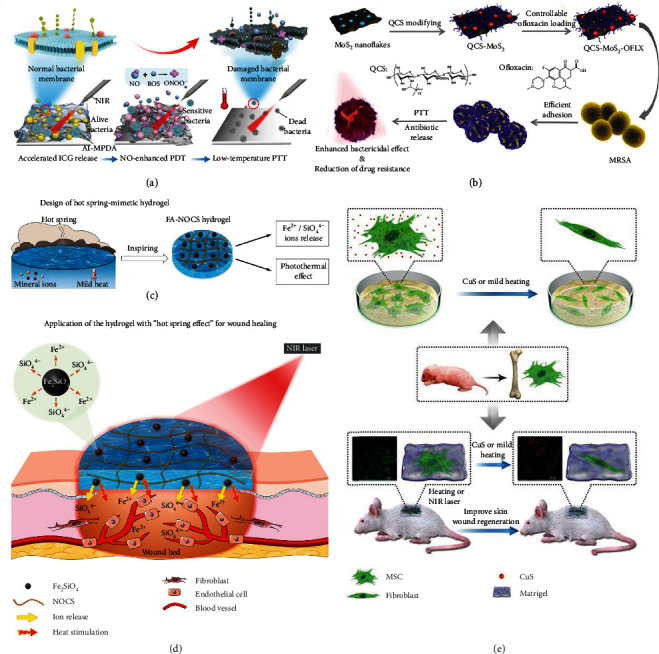
(a) Schematic illustration of NO-enhanced PDT and low-temperature PTT for biofilm eradication based on AI-MPDA NPs under NIR light irradiation. Reprinted with permission from Reference [59]. Copyright 2020, American Chemical Society. (b) Scheme of the synthesis of QCS-MoS_2_-OFLX and the synergistic low-temperature photothermal/antibiotic therapy against MRSA bacteria. Reprinted with permission from Reference [60]. Copyright 2020, Springer. (c) Design of hot spring-mimetic hydrogel. (d) Application of the FA-NOCS hydrogel with “hot spring effect” for wound healing. (c, d) Reprinted with permission from Reference [67]. Copyright 2021, Elsevier Ltd. (e) Scheme illustrating the differentiation of MSCs that were treated with CuS@BSA (in the presence or absence of NIR light irradiation) or mild heating into fibroblasts, and the skin wound closure function of these MSC-containing systems. Reprinted with permission from Reference [68]. Copyright 2020, Ivyspring.

One of the most frequently used nanomaterials for PTT-based antibacterial application is PDA, which is formed by the self-polymerization of dopamine. PDA has been applied extensively in the biomedical field because of its high biocompatibility and facile functionalization. Furthermore, PDA nanomaterials possessing fascinating NIR photothermal conversion efficiency are excellent PTAs that can address numerous issues including the treatment of bacterial infections. Hence, associating PDA with other components which can endow the assembled platforms with the capacity to facilitate the efficacy of low-temperature PTT has attracted tremendous attention. For instance, Fan et al. synthesized a novel nanoplatform composed of PDA NPs modified by magainin I (MagI), a typical antimicrobial peptide capable of preferentially interacting with the bacterial membrane, and thiolated poly(ethylene glycol) (PEG), a widely used biocompatible polymer employed to promote the NPs' dispersity and stability in hydrophilic environments [58]. After being irradiated by NIR light for only 500 s, the NPs elevated the temperature to 45°C in the suspension of *Escherichia coli* (*E*. *coli*) and presented exciting competence in targeting and killing bacteria. This work emphasizes that the close interaction between bacteria and PTAs can facilitate the effect of low-temperature PTT. Similarly, Yuan et al. reported a nanoplatform to get rid of the tough bacterial biofilm with nitric oxide- (NO-) enhanced PDT and low-temperature PTT via employing mesoporous PDA (MPDA) with surface modification of L-arginine (L-Arg) and further adsorption of indocyanine green (ICG) (the resultant product was termed AI-MPDA NPs) [59]. In the presence of NO synthase (NOS) existing in most cells, L-Arg could serve as a biocompatible donor of NO which has potential in interfering with bacterial DNA to disturb the bacteria's basic life activities. In the meantime, the reactive oxygen species (ROS) generated by ICG further oxidized NO to reactive peroxynitrite (ONOO^−^), consequently strengthening the PDT's efficiency, which further triggered the subsequent catalysis of L-Arg to release NO gas. Moreover, because NO-enhanced PDT could prominently disrupt the bacterial membranes to make the bacterial cells in biofilms sensitive to hyperthermia, low-temperature PTT induced by MPDA effectively led to the death of susceptible bacteria and the destruction of biofilms. Thus, after being injected to the bacteria-infected tissue where biofilms were already formed and being exposed to the NIR light, the AI-MPDA NPs residing in the focal area increased the temperature to 45°C and successfully killed bacteria and eliminated biofilms without eliciting notable inflammatory responses in vivo (Figure 2(a)). Further, the authors also demonstrated that AI-MPDA NPs could evoke a rapid wound healing effect.

Besides PDA, a two-dimensional molybdenum disulfide (MoS_2_) nanoflake (an NIR PTA) was also used by Huang et al. for defeating bacterial infections at mild temperatures [60]. The authors modified MoS_2_ nanoflakes with positively-charged quaternized chitosan (QCS) (yielding QCS-MoS_2_) to increase the dispersion stability and assist the MoS_2_ nanoflakes to adhere onto the bacterial surface more tightly. Further, the authors loaded ofloxacin, an antibiotic, onto QCS-MoS_2_. Under NIR light irradiation, the heat generated by QCS-MoS_2_ rendered methicillin-resistant *Staphylococcus aureus* (MRSA) more susceptible to ofloxacin at a low dose. The resulting nanoplatform (denoted as QCS-MoS_2_-OFLX) exhibited outstanding MRSA bacterium eradication effect, proving that the combined low-temperature PTT and antibiotic therapy could alleviate the drug resistance of bacteria (Figure 2(b)). Similarly, by employing red phosphorus NPs to realize low-temperature PTT, Tan et al. demonstrated that low-temperature photothermal treatment could make MRSA susceptible to conventional aminoglycoside antibiotics [61]. In this example, through exploring the underlying mechanisms, the authors proposed that low-temperature PTT owned the advantages of conquering multidrug-resistant (MDR) bacterial infection by restraining the activity of 2-aminoglycoside phosphotransferase. Thus, low-temperature PTT may pave a new way for the elimination of MDR bacteria and the improvement of the antibiotics' bactericidal effect.

### 2.2. Low-Temperature PTT for Promoting Wound Healing

In the past few decades, effectively promoting wound healing which is a common but complicated process in animals has been considered as an important task in the field of biomedical engineering. There exist several important problems in the wound healing issue, including bacterial contamination [62], chronic wound [63], and excessive wound healing [64]. Fortunately, with the deeper understanding of wound's pathophysiology and the development of biomaterials, a myriad of novel strategies have been proposed and diverse materials have been introduced into the wound healing therapeutic platforms. However, as shown in Table 1, up till now, only a few studies utilized low-temperature PTT to improve the effectiveness of wound therapy and they all exhibited satisfactory performance in facilitating wound healing [65–68].

For example, to conquer bacterial infection and alleviate surrounding inflammation for effective wound treatment, Xu et al. synthesized a gold NP-decorated hydroxyapatite (Hap) nanorod and modified the surface of the nanorod with PDA [65]. Through controlling the working time of NIR laser, the authors found that this nanoplatform could achieve low-temperature PTT for killing bacteria. Besides, this nanoplatform exhibited peroxidase-like activity and was capable of catalyzing hydrogen peroxide (H_2_O_2_) to produce highly reactive hydroxyl radical (·OH), thus rendering bacteria more sensitive to mild heat. Importantly, the release of Ca^2+^ and PO_4_^3−^ from Hap could facilitate the expression of tissue repairing-related genes and promote the formation of granulation tissue and collagen synthesis, which realized the rapid tissue regeneration and accelerated wound healing. Likewise, Li et al. fabricated a Hap/nitrogen-doped carbon dot- (NCD-) modified graphene oxide (GO) heterojunction (GO/NCD/Hap) film which showed the enhanced photocatalytic and photothermal effects [66]. Under NIR light irradiation, this film could not only achieve PDT and mild PTT for killing bacteria but also repair vascular injury via the Ca^2+^-activated PLC*γ*1/ERK pathway and avoid excessive inflammation by activating the PI3K/P-AKT pathway to promote wound healing. The above two studies both verify that the employment of low-temperature PTT can help to eliminate bacteria and facilitate wound healing, and demonstrate that Ca^2+^ plays a crucial role in regulating tissue regeneration.

Besides wounds with bacterial infections, there are other types of wounds like chronic wounds which also need to be treated with. Lately, it has been found that the presence of both appropriate mild heat and bioactive elements can elevate the vascular density of granulation tissue and achieve satisfactory wound healing promotion, which is known as “hot spring” effect [69]. Inspired by this fact, Sheng et al. prepared a new photothermal hydrogel using oxidized sodium alginate, *N*,*O*-carboxymethyl chitosan (NOCS), and fayalite (FA) composed of Fe^2+^ and SiO_4_^4−^ (Figure 2(c)) and evaluated the wound healing effect of this hydrogel by using full-thickness excisional wound model in diabetic mice [67]. Under mild NIR laser, the hydrogel showed satisfactory low-temperature photothermal effect and successfully released ferrous and silicate ions nearby the wound in vivo (Figure 2(d)). It was verified that the combination of mild heat and bioactive ions could promote endothelial cell proliferation and angiogenesis through activating different angiogenic factors and signaling pathways, finally realizing wound healing and tissue reconstruction. This example further emphasizes that some bioactive ions significantly contribute to wound healing and mild PTT can enhance the therapeutic effect of these bioactive ions. Therefore, the combination of bioactive ion-mediated treatment and low-temperature PTT may provide a new way for repairing chronic wounds under mild conditions.

Moreover, given that mesenchymal stem cells (MSCs) have the advantages of ease of isolation and expansion, great proliferative capability, multidirectional differentiation capacity, and immunomodulatory property [70, 71], MSC-based therapies have attracted increasing interest from many researchers working on the treatments of different kinds of diseases, like wounds [71] and cancer [72]. It has been found that both the MSC-induced vascular endothelial growth factor (VEGF) production and the MSC-involved collagen deposition can make contribution to skin tissue regeneration for wound healing [73]. Additionally, it is reported that copper has the potential in not only stimulating cell proliferation [74] but also boosting angiogenesis [75]. Recently, Xiao et al. used CuCl_2_·2H_2_O and bovine serum albumin (BSA) to construct ultrasmall CuS@BSA NPs via a biomineralization strategy in aqueous solution at a physiological temperature [68]. They isolated MSCs from the bone marrow of newborn mice. On the one hand, the CuS@BSA NPs showed the ability to induce MSCs to differentiate into fibroblasts. On the other hand, under NIR laser irradiation, the mild heat generated by CuS@BSA NPs which owned the photothermal conversion property also promoted the differentiation of MSCs. Furthermore, the authors encapsulated MSCs (preheated or not) with or without CuS@BSA NPs into Matrigel and seeded the mixture in the wound area to evaluate the wound healing efficacy of these therapeutic platforms. They found that in the presence of CuS@BSA NPs, both the MSCs preheated by a 42°C water bath and the MSCs in situ treated by mild heat generated by CuS@BSA NPs under NIR light irradiation showed the satisfactory capacity in differentiating into fibroblasts. The in vivo experimental results further demonstrated that the combination of CuS@BSA NPs and mild heating (preheating or in situ heating through NIR light irradiation) could enable MSCs to promote wound healing (Figure 2(e)). This work suggests that mild hyperthermia may be able to control the direction of MSC differentiation to some extent, and provides a novel strategy, i.e., the employment of low-temperature PTT, for improving MSC-based therapies for treating other diseases.

Although there are only limited studies paying attention to employing mild PTT to facilitate wound healing, we have obtained new insights into the synergistic utilization of low-temperature PTT and other therapeutic agents/strategies for wound treatments. These published examples may inspire more researchers to introduce other types of functional materials and alternative approaches into the mild PTT systems for more efficient and durable wound healing.

### 2.3. Low-Temperature PTT for Cancer Treatments

Cancer therapy has always been an extremely tough task since the advent of modern biology and medicine. In the past few decades, PTT for cancer treatments has achieved great improvement and showed good therapeutic performance with advantages of controllable administration, minimal invasiveness, and reduced side effects [1]. However, as mentioned above, there are diverse problems in desperate need to be solved in this region. Mild-temperature PTT has been recently recognized as a robust approach that can meet the requirement of defeating cancer with alleviated side effects. With the help of nanotechnology, more and more engineered therapeutic platforms can be orchestrated, such as those with stimulus-responsive characteristic for improving the therapeutic accuracy and those integrated with other functional molecules or motifs through a series of manufacturing methods for the accomplishment of multimodal cancer theranostics. As illustrated in Table 2 (which summarizes the common strategies for low-temperature cancer PTT) and Table 3 (which summarizes some typical examples that combine low-temperature PTT with other therapeutic modalities), there have been some smart and promising strategies to control the temperature during cancer PTT, which will be elaborated below.

#### 2.3.1. HSP Downregulation/Starvation Therapy

HSPs, a highly evolutionarily conserved group of chaperone proteins that can be observed in cells exposed to elevated temperatures, are found to be extremely important in the folding, maintenance of structural integrity, and appropriate regulation of a series of cytosolic proteins, especially under the stressful circumstances [76, 77]. Classified by their molecular mass with the kDa unit, HSPs are usually divided into small HSPs (molecular mass < 40 kDa), and the HSP60, HSP70, HSP90, and HSP100 families [47]. Unfortunately, in the past few decades, numerous studies have proven that HSPs, especially HSP90 and HSP70 families, can significantly help cancer cells cope with harsh environments, enabling the survival of cancer cells [76, 78]. Regarding HSP90 capable of maintaining the conformation, stability, and function of key client proteins related to oncogenic signal transduction (i.e., mutant epidermal growth factor receptor), angiogenesis (i.e., VEGF), antiapoptosis (i.e., AKT), and metastasis (i.e., matrix metalloproteinase 2 and CD91), processes important for maintaining the cancer phenotype, it is substantially expressed at 2- to 10-fold higher levels in tumor cells compared to their normal counterparts, suggesting that it may be crucial for tumor growth and/or survival [76, 79–81]. Similarly, in normal cells, proteins of the HSP70 family are mainly expressed under stressful conditions and there are also some constitutively expressed HSP70 proteins to maintain cellular homeostasis. In contrast to normal cells, it has been found that some proteins of HSP70 family are fundamentally overexpressed in malignant human tumor cells of various origins for tumor growth and survival [77, 82]. With its working mechanism being extensively investigated, HSP70 has been suggested to be highly relevant to apoptosis through not only interfering with the intrinsic pathway based on the successive activation of caspases [78] but also inhibiting caspase-independent apoptosis [82, 83] and lysosomal membrane permeabilization [84], resulting in the escape of cancer cells from programmed cell death. Consequently, the downregulation of HSPs has been regarded as an alternative strategy for cancer treatment, and plenty of studies have suggested that the inhibition of HSPs can give rise to tumor suppression [85–87]. Since hyperthermia can trigger the overexpression of HSPs, which is the vital reason for the thermoresistance of cancer cells, it is reasonable to associate HSP downregulation with PTT to achieve high-performance low-temperature PTT. Inspiringly, the prosperity of nanotechnology has pushed HSP inhibition-mediated mild PTT to an unprecedented climax. Herein, we divide the well-known strategies of HSP suppression into two types, i.e., the use of HSP inhibitors and cancer starvation therapy, and will introduce the corresponding action mechanisms and related advancements in the following parts.

*(1) HSP Inhibitors*. With a deeper understanding of HSPs at the cell and molecular biology level, researchers encountered the advent of some highly specific HSP inhibitors and successfully employed them to experiments. For example, the benzoquinone ansamycin geldanamycin and 17-allylaminogeldanamycin were identified and put into clinical trials many years ago [88, 89]. Lately, scientists also found several other HSP inhibitors, represented by gambogic acid (GA), and figured out the mechanism at the molecular level [81, 90]. GA, a universally used HSP90 inhibitor which can weaken thermoresistance and induce apoptosis of cancer cells, has been applied in clinical and preclinical trials. With the development of genomics, small interfering RNA (siRNA) HSP inhibitors exerting their activity on transcription have attracted considerable attention [91, 92]. However, similar to chemotherapeutic drugs, the utility of HSP inhibitors still faces some problems, such as wide body distribution, low bioavailability, and unsatisfactory pharmacokinetics. To cope with the dilemma, as mentioned above, changing the formulation of a delivery system through nanotechnology and combining HSP inhibitors with other treatment methodologies have shed new light on these issues.

**Figure 3:**
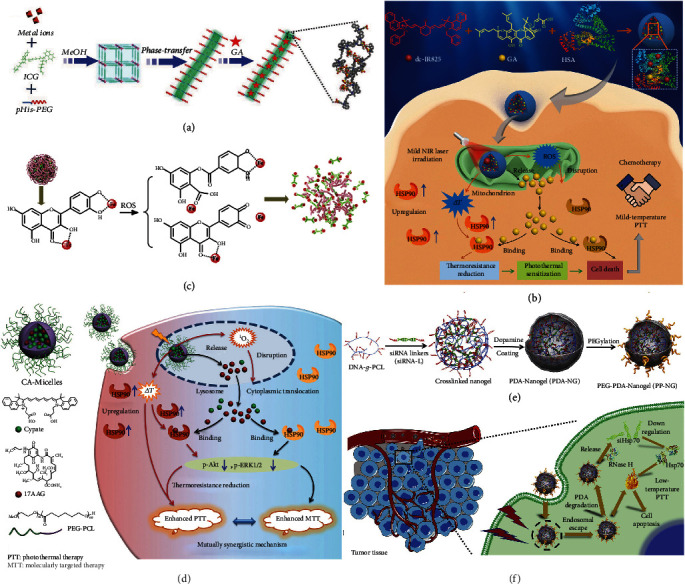
(a) Scheme illustrating the one-step synthesis of one-dimensional coordination polymer nanofibers. Reprinted with permission from Reference [93]. Copyright 2017, Wiley-VCH. (b) Fabrication and working mechanism of HSA/dc-IR825/GA NPs for synergistic molecular targeting-mediated mild-temperature PTT and chemotherapy. Reprinted with permission from Reference [27]. Copyright 2019, Wiley-VCH. (c) Mechanism of the ROS-mediated disassembly of Qu-Fe^II^Ps. Reprinted with permission from Reference [99]. Copyright 2019, Elsevier Ltd. (d) Scheme showing the structure and components of the CA-micelles and their use for mutually synergistic MTT/PTT. Reprinted with permission from Reference [101]. Copyright 2017, Wiley-VCH. (e) Schematic illustration of the synthesis of PP-NGs. (f) Scheme showing the mechanism of low-temperature PTT realized by PP-NGs. (e, f) Reprinted with permission from Reference [108]. Copyright 2020, Elsevier Ltd.

*Small-Molecule HSP Inhibitors*. Up till now, scientists have taken advantage of dozens of the existing nanomaterials to endow mild-temperature PTT platforms containing small-molecule HSP inhibitors with increased drug loading capacity and decreased nonspecific cytotoxicity. For example, Yang et al. proposed a facile method to synthesize a PEGylated one-dimensional coordination polymer via a one-step reaction and phase transfer [93]. ICG (an NIR dye), poly-L-histidine-PEG (pHis-PEG), and Mn^2+^ (a frequently used contrast agent in magnetic resonance (MR) imaging, MRI) were first self-assembled to build a three-dimensional porous framework in the methanol solution via coordination interaction, and after being transferred into the aqueous solution, the three-dimensional porous materials finally became one-dimensional nanofibers. Then, the authors integrated GA into the nanofibers to afford Mn-ICG@pHis-PEG/GA (Figure 3(a)) and uncovered that this nanoplatform could prominently facilitate cancer cell apoptosis and realize efficient low-temperature PTT. Moreover, the nanoplatform showed enhanced cell internalization and tumor retention due to its pH-responsive ability, which was attributed to the protonation of imidazole groups in pHis-PEG under the acidic environment. Recently, our group established an intelligent molecular targeting-mediated nanoplatform composed of GA, dc-IR825 (a fluorescent probe and a PTA), and human serum albumin (HSA) (a biocompatible carrier for hydrophobic molecules) [27]. The nanoplatform (HSA/dc-IR825/GA NPs) showed intrinsic low pH-induced charge reversal which was beneficial for the notably increased cellular uptake and tumor accumulation under the acidic tumor microenvironment (TME). It was found that the NPs were well located in mitochondria after endocytosis. Under NIR light irradiation, the ROS generated by dc-IR825 in the NPs effectively destructed mitochondrial membranes, which resulted in the escape of GA from the mitochondria to the cytoplasm and subsequent HSP90 inhibition for low-temperature PTT (Figure 3(b)). Additionally, we have also demonstrated that the nanoplatform could realize synergistic low-temperature PTT/GA-mediated molecularly targeted therapy (MTT) in vivo and efficiently eliminate tumors and inhibit tumor metastasis. In another example, Li et al. fabricated zeolitic imidazole framework-8 (ZIF-8) NPs and embedded bismuth nanodots in the ZIF-8 NPs (abbreviated as BZ) by a simplified one-step reduction method [94]. This composite nanomaterial possessed satisfactory photothermal conversion efficiency in the NIR-II (1000–1350 nm) region, which was important for realizing deep tissue penetration. The authors further loaded GA onto BZ to afford GBZ, and demonstrated that GBZ could achieve markedly accelerated GA release under acidic conditions and NIR light exposure. Moreover, the authors revealed that GBZ could successfully treat hepatocellular carcinoma in mouse models. These studies all emphasize that GA-containing nanoplatforms can remarkably alleviate the thermal resistance of cancer cells and induce cancer cell apoptosis for enhanced low-temperature PTT. Moreover, the low pH-responsive property of nanomaterials is crucial for realizing specific tumor accumulation and/or GA release, which may inspire researchers to design more smart nanoagents with multiple exogenous and/or endogenous stimulus-responsive capacity for improving the performance of low-temperature PTT systems.

Recently, Sun et al. proposed a novel one-pot synthesis method for fabricating hollow mesoporous carbon spheres (HMCSs), which had the eminent potential in photothermal conversion and photoacoustic (PA) imaging (PAI) [95]. After PEG modification and GA loading, the resultant HMCS-PEG-GA displayed its capacity in ablating tumors under moderate temperature environments controlled by the NIR laser. Semiconducting polymer NPs and black phosphorus quantum dots have also been employed to encapsulate GA for promoting the efficiency of PTT under mild NIR light irradiation [96, 97]. In addition, the Chen group fabricated a scaffold to achieve low-temperature PTT of breast cancer. They first synthesized poly(acrylic acid)-*g*-poly(lactic acid) (PAA-*g*-PLLA) and then connected PAA-*g*-PLLA with graphene oxide (GO) via a low-pH cleavable bond, forming GO-PAA-*g*-PLLA [98]. After GA was loaded to GO-PAA-*g*-PLLA, the obtained complex could release GO/GA in the acidic TME due to the low-pH cleavable bond for mild PTT. Further, poly(caprolactone) (PCL) was employed as the framework to form the final GO-GA-polymer scaffold via mixing PCL with GA-loaded GO-PAA-*g*-PLLA. The scaffold-mediated low-temperature PTT could effectively achieve tumor eradication both in vitro and in vivo. The authors then inserted adipose-derived stem cells into the interspace of the scaffold to deliver the stem cells for adipose tissue regeneration at the site of breast cancer, finally realizing reconstruction of breasts after surgical resection. This work provides a promising strategy for the combination of tumor ablation and tissue regeneration under gentle hyperthermia.

Besides GA, there are many other small-molecule HSP inhibitors that have been employed in low-temperature PTT for achieving satisfactory cancer treatment outcomes. For example, Yang et al. employed quercetin (Qu) as a multifunctional element to connect with ferrous iron (Fe^II^) and polyvinylpyrrolidone (PVP) via coordination interaction to prepare nanodrugs (denoted as Qu-Fe^II^Ps) [99]. Qu had several important roles in this nanosystem: First, it is an inhibitor of HSP70 for realizing mild hyperthermia under NIR light irradiation [100]. Second, since quercetin possesses good ROS-scavenging ability, the Qu-Fe^II^Ps could clear excessive ROS which might endanger surrounding healthy tissues, and reduce ROS-induced inflammatory cytokines to avoid unexpected inflammation [99]. Meanwhile, the coordination between Qu and Fe^II^ became loose when Qu was oxidized by ROS, and subsequently Qu-Fe^II^Ps were disassembled into ultrasmall NPs (Figure 3(c)), which led to rapid renal clearance of the NPs to minimize acute or chronic toxicity. This study highlights the advantage of using Qu for constructing nanomaterials for mild PTT.

17-Allylamino-17-demethoxygeldanamycin (17-AAG, also termed tanespimycin), another common HSP90 inhibitor, has also been applied for constructing low-temperature PTT systems. For example, Luo et al. loaded 17-AAG into a well-designed micelle (named as CA-Micelle) composed of PEG_114_-PCL_60_ (in which PCL is the abbreviation of poly(*ε*-caprolactone)) and cypate, one of the cyanine dyes capable of yielding hyperthermia under NIR light irradiation [101]. Because cypate could also produce ROS under NIR light irradiation, the micelles achieved the cytoplasmic translocation of 17-AAG to interact with HSP90 by ROS-mediated ysosomal disruption. It was found that 17-AAG was capable of inhibiting antiapoptotic p-ERK1/2 proteins to promote early apoptosis for effective 17-AAG-mediated MTT and downregulating the expression of p-Akt to attenuate thermoresistance of cancer cells for realizing promoted low-temperature PTT (Figure 3(d)). Moreover, the late apoptosis was also enhanced due to the combination of PTT and MTT, thus leading to excellent tumor eradication effect. In another example, the Qian group entrapped VER-155008 which can restrict the activity of both HSP70 and HSP90 into methoxy PEG-poly(D,L-lactic acid) to form VER-155008 micelle to improve the water solubility of VER-155008 [102]. Then, this VER-155008 micelle was mixed with methoxy PEG-coated gold nanorod (AuNR) (serving as a PTA) to achieve the enhanced tumor depletion under mild temperature. In addition, 2-phenylethynesulfonamide (PES), famous for disturbing HSP70 activity via multiple cell signaling pathways with excellent specificity and negligible cytotoxicity, was introduced into a poly(3,4-ethylenedioxythiophene)- (PEDOT-) encapsulated thermoresponsive nanogel composed of poly(*N*-isopropylacrylamide-*co*-acrylic acid) (PNIPAM) by Liu et al. [103]. To meet the requirements of real-time diagnosis, Jiang et al. fabricated a flower-like NiS_2_-coated NaLuF_4_:Nd (Lu:Nd@NiS_2_) NP for short-wave infrared light imaging and MRI-guided PTT through using epigallocatechin gallate (EGCG) that can suppress HSP90 activity [104]. This NP showed satisfactory antitumor efficacy via low-temperature PTT and could be employed as an outstanding theranostic nanoplatform. Recently, Zhang et al. adopted BIIB021, an HSP90 inhibitor, to prepare a novel mild-temperature PTT platform by first synthesizing PEG-IR780 (IR780 is an NIR cyanine dye) via covalent conjugation between PEG-SH and IR780 and then adding BIIB021 to obtain PEG-IR780-BIIB021 nanomicelles via the solvent evaporation method [105]. The authors demonstrated that the positively charged NIR PTA IR780 could selectively accumulate in cancer cell mitochondria where the membrane potential is higher than that of normal cell mitochondria, thus realizing effective mitochondrion-targeted cancer therapy. Moreover, the nanomicelles located in the mitochondria of the cancer cells generated heat and released BIIB021 to reduce the tumor cell resistance to mild hyperthermia after NIR light irradiation, leading to the decrease of mitochondrial membrane potential and quick release of crucial intrinsic apoptotic factors to activate the mitochondrial apoptotic pathway for achieving high-performance low-temperature PTT. Due to the potential of BIIB021 in conquering breast cancer possessing overexpressed HSP90, this nanomicelle exhibited promising capacity for mild PTT of breast cancer.

The above examples highlight that the integration of small-molecule HSP inhibitors (including GA, Qu, 17-AAG, VER-155008, PES, EGCG, and BIIB021) into PTT nanoplatforms represents an important strategy to deepen our understanding of the working mechanism of PTT and achieve the safe and effective tumor ablation via low-temperature PTT.

*siRNA HSP Inhibitors*. In the past few years, siRNAs have shown great potential for low-temperature PTT because some of them can downregulate HSPs at the genetic level through gene silencing. The siRNAs against HSPs are capable of interacting with the messenger RNAs (mRNAs) of HSPs via complementary base pairs, and then, RNA-induced silencing complexes are formed to cut mRNAs into small pieces, thus inhibiting the expression of HSPs [106–108]. However, there are diverse biomolecules including nucleases in the blood stream able to degrade naked nucleic acids during the delivery process [109]. Furthermore, nucleic acids must escape from endosomes before the endosomes fuse with lysosomes containing various enzymes that can inactivate or degrade foreign molecules. To this end, Wang et al. embedded siRNAs against HSP70 into the pores of cypate-conjugated upconversion nanocomposites for mild photothermal ablation of tumors with the assistance of upconversion luminescence imaging and MRI [106]. Given that gold nanostructures possess advantages of versatile nucleic acid conjunction, satisfactory photothermal conversion efficiency, and acceptable biosafety, Wang et al. utilized gold to construct nanoshells, which were modified with siRNAs against HSP70 to make the cancer cells more susceptible to moderate hyperthermia [107]. In another example, Ding et al. fabricated an anti-Hsp70 siRNA (siHsp70) delivery platform, which possessed three shields to protect siRNA [108]. First, siHsp70 and DNA-grafted PCL (DNA-*g*-PCL) were assembled into nanogels by nucleic acid hybridization. Second, PDA was employed to form a thin layer attached on the surface of the above-obtained nanogels, which introduced photothermal conversion capability to the whole nanoplatform. Third, after surface PEGylation, the nanoplatform (denoted as PP-NG) (Figure 3(e)) displayed elevated physiological stability and inhibited the degradation of siRNAs by ribonuclease (RNase) A. Moreover, it was found that in the acidic environments of endosomes or endolysosomes which encapsulated PP-NGs, the oligomer PDA would degrade and detach from the PDA layer of PP-NGs, thus facilitating the release of nanogels from PP-NGs and inducing the nanogels to be exposed to the enzymes. Further, under NIR light irradiation, the PDA-mediated hyperthermia could help the nanogels escape from endolysosomes to the cytoplasm where they could be degraded by RNase H (which recognizes DNA-RNA hybrid duplex and digests the RNA strand), and then, siRNAs were released to the cytoplasm to downregulate HSP70 to realize effective low-temperature PTT (Figure 3(f)). This work exemplifies a feasible solution to realize safe siRNA delivery for low-temperature PTT.

*(2) Cancer Starvation Therapy*. Lately, cancer starvation therapy has been introduced for the ablation of malignant tumors [110]. As is well known, tumors display fast nutrition consumption to maintain the rapid speed of cancer cells' growth and proliferation. On the one hand, cancer cells secrete several proangiogenic growth factors and express the counterpart receptors of the factors. Therefore, there are abundant blood vessels around and inside tumors that ensure the tumors' sufficient nutrition supply for their survival and growth. On the other hand, cancer cells tend to obtain energy by an abnormal type of metabolism (the Warburg effect) that mainly sustains energy supply through anaerobic glycolysis, which exacerbates their reliance on glucose [111]. Hence, two major principles of designing platforms for cancer starvation therapy have been proposed [110]: (1) inhibiting angiogenesis or blocking tumor blood vessels and (2) reducing the cellular uptake of or exhausting life-necessary nutrients, especially glucose. Unfortunately, starvation therapy only partially succeeds due to a series of obstacles like increased tumor hypoxia, unsatisfactory targeting capacity, induced drug resistance, and elevated tumor metastasis risk, which all impede their further applications in clinic [110]. To overcome these challenges (or some of these challenges), combining starvation therapy with PTT serves as a good choice since it can maximize the anticancer therapeutic efficiency [110, 112]. More importantly, given that both the production of HSPs and the execution of their bioactive processes need ATP, developing strategies that can lead to the lack of ATP via blocking or decreasing the supply of energy sources like glucose provides an efficacious way to realize HSP inhibition and hence mild-temperature PTT. Therefore, cancer starvation therapy represents one promising strategy that can be combined with low-temperature PTT to achieve potentiated anticancer effect.

**Figure 4:**
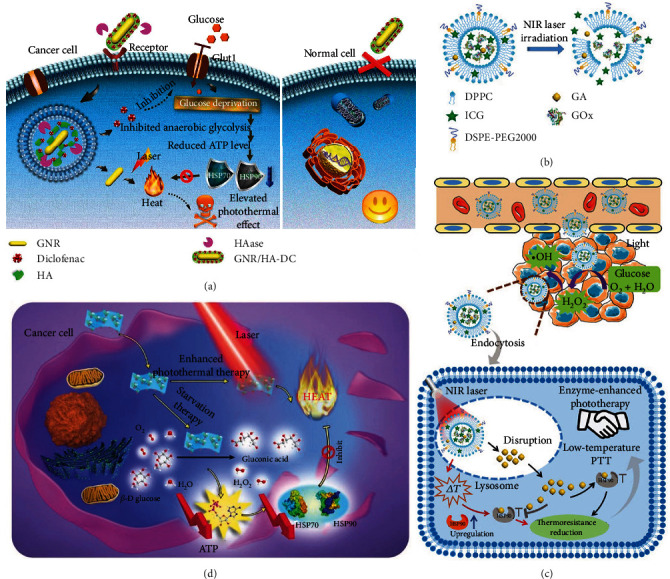
(a) Scheme showing the use of GNR/HA-DC to sensitize cancer cells to mild PTT by interfering with the anaerobic glycolysis metabolism. Reprinted with permission from Reference [113]. Copyright 2017, American Chemical Society. (b) Scheme illustrating the transformation of GOIGLs upon NIR light irradiation. (c) Scheme depicting the mechanism of EEPT and low-temperature PTT. (b, c) Reprinted with permission from Reference [32]. Copyright 2020, Wiley-VCH. (d) Scheme showing the utilization of M-NS for cancer starvation therapy and PTT. Reprinted with permission from Reference [119]. Copyright 2019, Wiley-VCH.

Considering that there are overexpressed glucose transporters on the membranes of tumor cells, blocking or downregulating these transporters acts as an emerging solution to deprive cancer cells of cytoplasmic glucose for cancer starvation therapy. This strategy has been employed for enhanced mild PTT. For example, Chen et al. used diclofenac (DC), which served as a small-molecule inhibitor of Glut1 (one of the major glucose transporters (Gluts)), and hyaluronic acid- (HA-) modified plasmonic gold nanorod (GNR) to construct GNR/HA-DC for realizing low-temperature PTT [113]. As some cancer cells overexpress CD44 receptors capable of interacting with HA and TME is highly abundant in hyaluronidase (HAase), this platform could target CD44-overexpressed tumors and achieve the HAase-mediated release of DC to the cytoplasm to inhibit Glut1. Thanks to its surface plasmon resonance property, GNR/HA-DC exhibited noticeable photothermal conversion capacity, which was beneficial for realizing cancer-specific mild-temperature PTT especially for CD44-overexpressed tumor cells (Figure 4(a)).

Moreover, glucose oxidase (GOx), which can catalyze glucose, O_2_, and H_2_O into gluconic acid and H_2_O_2_, has been widely used in cancer therapy [114–116]. In the past few years, due to the ability of GOx to achieve cancer starvation, some researchers utilized GOx to realize low-temperature PTT of tumor [32, 117, 118]. For instance, a nanoreactor was prepared by Cao et al. to combine tumor starvation therapy with multiple mechanism-enhanced mild-temperature phototherapy for cancer treatment [117]. They loaded both GOx and ICG onto the Fe-doped polydiaminopyridine (Fe-PDAP) nanozyme via electrostatic adsorption and *π*-*π* interaction to obtain the Fe-PDAP/GOx/ICG nanoreactor. On the one hand, the Fe-PDAP nanozyme could catalyze H_2_O_2_ produced during GOx-mediated glucose consumption as well as supplied by tumor cells into O_2_ to improve O_2_-dependent ICG-based PDT. On the other hand, the Fe-PDAP nanozyme was capable of depleting GSH (which is the key antioxidant agent capable of reducing ROS) by a metal-reducing reaction after releasing Fe^3+^, thus further enhancing the efficiency of PDT. Meanwhile, under NIR light irradiation, mild PTT was achieved due to GOx-mediated HSP downregulation and ICG-based hyperthermia. To be noted, the Fe-PDAP/GOx/ICG showed satisfactory performance in multimodal fluorescence imaging (FLI)/PAI/MRI. This work verifies that the GOx-mediated cancer starvation therapy has advantages in not only downregulating HSPs for low-temperature PTT but also remarkably improving the synergistic cancer therapeutic efficiency. However, protecting GOx from deterioration and leakage during the circulation and endocytosis processes to execute catalysis validly is a disturbing problem. To deal with this issue, researches have designed a variety of GOx-containing nanomaterials and further optimized the GOx release process by the introduction of stimulus responsiveness [32, 118]. Zhou et al. entrapped GOx into a porous hollow Prussian blue (PB) NP (termed PHPBN) which is a biologically friendly NIR PTA and can protect GOx from being inactivated [118]. Further, they conjugated the surface of GOx-loaded PHPBNs with HA through disulfide bonds, which can be cleaved by intracellular glutathione (GSH) to realize the GOx release into the cytoplasm, and meanwhile, the HA can help the nanoplatform to target the CD44 receptors overexpressed on the plasma membranes of HepG2 cells. Finally, PEG was grafted to the outer shell to prolong the blood circulation time. Importantly, the PHPBNs were able to catalyze H_2_O_2_ generated during GOx-mediated glucose oxidation to produce oxygen for alleviating tumor hypoxia and elevating the catalytic efficacy of GOx, finally achieving effective cancer starvation therapy and low-temperature PTT. In another example, our group prepared thermosensitive liposomes which were loaded with GA, GOx, and ICG to afford GOIGLs [32]. Upon NIR light irradiation, ICG within the liposomes could efficiently convert light to heat, and when the temperature was elevated to above 42°C, the liposomes underwent the gel-to-fluid phase transition and subsequently released the loaded GA, GOx, and ICG (Figure 4(b)). Additionally, under visible light illumination (400–750 nm), H_2_O_2_ which was produced during the GOx-catalyzed glucose oxidation reaction could be transformed into ·OH, which is one of the most lethal ROS to cells, thus showing enzyme-enhanced phototherapy (EEPT) effect. Besides, after NIR light irradiation, the GOIGLs could successfully escape from lysosomes via ROS-mediated lysosomal disruption, which contributed to the release of GOx and GA to the cytoplasm to consume glucose and downregulate HSPs, respectively, thus realizing synergistic starvation therapy and low-temperature PTT (Figure 4(c)). This work emphasizes the robust anticancer therapeutic effect of the combined use of HSP inhibitor-promoted mild PTT and GOx-mediated cancer starvation therapy. Besides GOx, GOx-like nanoagents can also be used to construct the starvation therapy/mild PTT-based platforms for defeating cancer. As an example, Tang et al. fabricated a two-dimensional MnO_2_ nanosheet (M-NS) through a unique one-step wet-chemical method, which could control the size and thickness of M-NSs by adjusting the ratio of BSA to MnCl_2_ in oxygen-containing alkaline solutions [119]. Moreover, the M-NSs exhibited excellent GOx-like catalytic performance and NIR-absorbing ability. Thus, the M-NSs could consume intracellular glucose leading to the downregulation of HSPs and generate heat under NIR light irradiation, finally achieving low-temperature PTT (Figure 4(d)). The authors also developed a novel sono-chemical way to coat the surface of M-NSs with 2-*S*-(4-isothiocyanatobenzyl)-1,4,7-triazacyclononane-1,4,7-triacetic acid-modified BSA (BSA-NOTA) and found that the modified M-NSs possessed improved stability and accessibility of further functionalization, such as chelation with radioactive ^64^Cu for positron emission tomography imaging. The above reports all utilize the advantages of GOx or its mimics to construct cancer therapeutic nanoplatforms and emphasize the eminent potential of GOx/GOx mimics-mediated tumor starvation in promoting effective low-temperature PTT.

Besides employing the Glut inhibitors and GOx, Dang et al. exploited a new way to accomplish synergistic cancer starvation therapy/low-temperature PTT. The authors used an siRNA against pyruvate kinase M2 (siPKM2, which is capable of interfering with tumor glycolysis) and ICG to fabricate a novel nanocomplex based on a spherical dendrimer polypeptide (DPP) [120]. Specifically, amine-terminated polyamidoamine (PAMAM) was utilized as a macroinitiator template to synthesize DPP via controlled ring-opening polymerization (ROP) of *N*-carboxyanhydride followed by side-chain functionalization with guanidines. The DPP could load ICG into its hydrophobic cavity, and meanwhile, siPKM2 was condensed by DPP via electrostatic interaction to form positively charged nanocomplexes. Further, to improve the stability and biosafety, the nanocomplexes were enveloped by human serum albumin to obtain the final product termed D-I/P@HSA NCs (NCs: nanocomplexes). Under NIR light irradiation, the NCs exhibited enhanced tumor photothermal ablation efficiency by the aid of the siPKM2-mediated cancer starvation effect. This research points out that disturbing tumor glycolysis via molecular or nanoscale agents may represent a novel approach to realize the combination of cancer starvation therapy and low-temperature PTT.

#### 2.3.2. Autophagy-Mediated Cancer Therapy

Autophagy, an essential catabolic process, can degrade and recycle misfolded proteins or damaged organelles to conserve cellular homeostasis [121]. Nevertheless, there have been arguments about the role of autophagy in the occurrence and progression of cancers. On the one hand, autophagy can accelerate tumor development and shield cancer cells from diverse cellular stresses like chemical drugs and radiation, eventually making tumors develop chemo- and radioresistance and be difficult to be cured [122–125]. On the other hand, autophagy can restrict tumorigenesis via regulating many oncogenes and tumor suppressor genes and inhibit tumor invasion and metastasis from the primary sites via limiting necrosis and inflammation [122–124]. Thus, both the promotion and inhibition of autophagy have been applied to defeat cancers.

**Figure 5:**
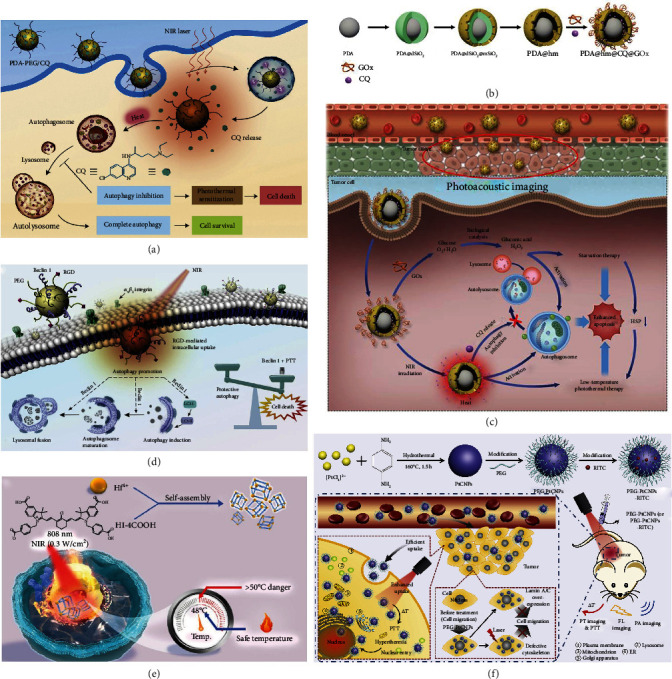
(a) Scheme of the enhanced cancer therapeutic efficiency of mild PTT resulting from photothermal sensitization through CQ-mediated autophagy inhibition. Reprinted with permission from Reference [129]. Copyright 2017, Elsevier Ltd. (b) Multistep preparation process of the rattle-structured PDA@hm@CQ@GOx NP. (c) Schematic illustration of the working mechanism of PDA@hm@CQ@GOx NPs that can achieve enhanced low-temperature PTT under NIR light irradiation. (b, c) Reprinted with permission from Reference [131]. Copyright 2020, Ivyspring. (d) Scheme depicting the beclin 1-induced autophagy rendering cancer cells vulnerable to photothermal elimination. Reprinted with permission from Reference [133]. Copyright 2019, Elsevier Ltd. (e) Scheme illustrating the Hf-HI-4COOH NCP structure and nucleus-targeted low-temperature PTT. Reprinted with permission from Reference [142]. Copyright 2020, Elsevier Ltd. (f) Scheme depicting the synthesis of PEG-PtCNPs and PEG-PtCNPs-RITC and their application for multimodal imaging-guided and multiorganelle-targeted PTT. Reprinted with permission from Reference [145]. Copyright 2018, Elsevier Ltd.

Given that inhibition of autophagy may contribute to tumor eradication, researchers have recently invented many approaches to inhibit autophagy for improved cancer treatments [126–128]. Among these approaches, the combination of autophagy-modulating molecules and mild PTT shows the satisfactory ability of eliminating tumors at gentle temperatures [129–132]. Some studies have figured out that low-temperature PTT can induce both apoptosis and autophagy in cancer cells, and autophagy provides a survival way for malignant tumors [129, 130]. Further, it has been revealed that the inhibition of autophagy can augment apoptosis and apparently strengthen the efficiency of PTT, verified by the lifted malignant cells' fatality under mild hyperthermia using the autophagy inhibitor chloroquine (CQ) [129] and chloroquine diphosphate-loaded NPs [130]. It is noteworthy that the research carried out by Zhou et al. is the first report of using CQ-mediated autophagy inhibition to enhance PTT [129]. The authors loaded CQ on the surface of PEG-modified PDA NPs (the resultant product was termed PDA-PEG/CQ NPs) and found that CQ could be released from PDA NPs in the moderately acidic environment (pH = 6). The authors demonstrated that this nanoplatform could realize effective CQ release in the TME and cytoplasm to inhibit autophagy, leading to the elevated cancer cell sensitization to mild PTT for defeating cancer (Figure 5(a)). Lately, Shao et al. synthesized a rattle-structured NP by encapsulating a PDA nanocore in a hollow mesoporous silica nanoshell to afford PDA@hm [131]. Then, CQ was loaded into the hollow cavity of PDA@hm and GOx was conjugated onto the surface of the above obtained NP, thus obtaining PDA@hm@CQ@GOx (Figure 5(b)). It was demonstrated that this nanoplatform could successfully achieve enhanced photothermal eradication of tumors under the moderate temperature with the assistance of GOx-mediated starvation and CQ-mediated autophagy inhibition, consequently reducing cancer survival rates (Figure 5(c)). Additionally, the CQ-mediated autophagy inhibition made contribution to compensating for the loss of mild PTT and starvation therapy efficacies due to PTT- and starvation-activated autophagy, showing the outstanding synergistic effect of the PDA@hm@CQ@GOx. Moreover, the nanoplatform was capable of realizing PAI. This trimodal synergistic treatment strategy (i.e., autophagy inhibition, starvation therapy, and mild PTT) has a great potential in overcoming PTT's side effects and elevating the efficiency of low-temperature PTT. Recently, to break the cycle between tumor cell proliferation and bone resorption (this cycle plays an important role in the promotion of bone tumors' progression and metastasis), Wang et al. fabricated PEG-conjugated alendronate-functionalized and CQ-loaded PDA NPs (PPA/CQ) for bone cancer therapy [132]. It was found that, owing to the alendronate, PAA had the advantage of strongly interacting with hydroxyapatite abundant in bone tissues, thus realizing the bone-targeting function. Besides facilitating the PDA-mediated photothermal ablation of bone tumors at mild temperatures, CQ could also attenuate tumor-associated bone resorption via interfering with the differentiation and activation of osteoclasts and subsequently inhibiting osteoclastogenesis. The in vivo experiments showed that the PPA/CQ nanoplatform achieved both tumor elimination and osteolysis inhibition. This work verifies that CQ-mediated autophagy inhibition provides a feasible method for malignant bone tumor treatments.

On the other hand, the augment of autophagy in cancer cells has also been exploited to be combined with mild PTT for defeating tumors. Zhou et al. fabricated a PEGlyated melanin-like PDA NP decorated with a cyclic Arg-Gly-Asp (RGD) peptide which is famous for binding integrin overexpressed in tumor tissues, and a beclin 1-derived peptide (denoted as beclin 1) which is the product of a putative gene related to the upregulation of autophagy [133]. As expected, the NPs showed tumor-targeting capability and enhanced low-temperature PTT efficiency (resulting from the promotion of autophagy) (Figure 5(d)).

Collectively, the above examples highlight that we must pay more attention to figuring out and utilizing the complicated relationship between mild PTT and autophagy regulation to elevate the efficiency of tumor eradication.

#### 2.3.3. Organelle-Targeting Strategy

Undoubtedly, the integrity of the structure and function of all cellular organelles is the crucial support for cell growth and proliferation, and it is also of great significance in the invasion and metastasis of cancer cells [134, 135]. It is worth noting that a large number of studies have attached great importance to developing biomedical engineering platforms with awesome organelle-targeting capacity for bothering or even devastating the organelles and finally deteriorating tumors [24, 25, 30, 126, 136–139]. Most of the organelles are very susceptible to elevated temperatures, especially the cell nucleus enveloping approximately all the genetic materials easy to denaturation, which brings hope to low-temperature PTT. On the other hand, there are numerous pores consisting of nuclear pore complexes (NPCs) on the nucleus envelop, playing an important role in facilitating substance transportation and signal conduction between the nucleus and cytoplasm. Numerous studies spared no effort to develop highly reliable and effective drugs or delivery systems possessing the capability of taking action in the nucleus following passing through these tiny pores. For instance, Cao et al. engineered a cell nucleus-targeting TAT peptide onto the surface of a vanadium carbide quantum dot to obtain V_2_C-TAT, which could serve as a robust NIR-II PTA [140]. After encapsulating V_2_C-TATs into RGD peptide-decorated endogenous exosomes which were beneficial for the elevated biocompatibility, nonimmunogenicity, and long blood circulation ability, the authors found that the nanosystem could gather nearby the tumor and enter the nucleus to destroy the genetic materials directly for low-temperature PTT with the dual guidance from PAI and MRI. Similarly, our group found that the palladium nanosheets modified by TAT (Pd-TAT) mainly accumulated in the perinuclear region and showed increased endocytosis and reduced efflux [14]. Importantly, Pd-TAT in the perinuclear region could trigger the overexpression of lamin A/C proteins to elevate nuclear stiffness and inhibit cancer metastasis, because lamin A/C proteins are highly related to nuclear stiffness and lack of the proteins facilitates cell migration. Under mild NIR light irradiation, the Pd-TAT presented perinuclear-to-intranuclear translocation and exhibited enhanced cancer cell metastasis inhibition and low-temperature PTT effect. In addition, Liu et al. synthesized an ultrasmall chitosan-coated ruthenium(IV) oxide NP with excellent NIR-II photothermal conversion ability and suitability for PAI [141]. The NPs could accumulate in the cell nucleus and release mild heat enough for destroying nuclear DNA under NIR-II light irradiation. Jiang et al. synthesized an Hf-heptamethine indocyanine dye- (Hf-HI-4COOH-) based nanoscale coordination polymer (NCP) which possessed strong NIR absorption and excellent photothermal conversion capacity to serve as an intrinsic nucleus-targeting PTA [142]. Under low-power NIR light irradiation, the NCP could generate enough heat to destroy the nucleus at a mild temperature, leading to the elimination of cancer cells. This study expands the application of NCPs and provides a new choice for conducting nucleus-targeted low-temperature PTT (Figure 5(e)).

Additionally, mitochondrion which is considered as the cellular energy factory and apoptosis control center has been regarded as a key location where mild hyperthermia can achieve a satisfactory cancer cell killing effect. Based on this, Qiu et al. constructed mitochondrion-targeting lipophilic iridium(III) cation-modified Fe_3_O_4_ NPs [143]. The NPs not only showed superb MRI/photothermogenic ability but also worked as a nanozyme for executing the Fenton reaction by catalyzing H_2_O_2_ generated from the mitochondrial respiratory electron transport chain (this catalysis process could be stimulated by the heat stress of mild PTT) into ·OH for killing cancer cells, thus realizing excellent MRI-guided and nanozyme-mediated mild-temperature PTT. In addition, the cytoskeleton, which is highly related to tumor migration, is a proper target for a variety of therapeutics [144, 145]. Our group fabricated platinum-doped carbon NPs (CNPs) by a facile one-pot hydrothermal method and modified them with PEG to afford PEG-PtCNPs (Figure 5(f)) [145]. To observe the behaviors of PEG-PtCNPs both in vitro and in vivo, we conjugated rhodamine B isothiocyanate (RITC) onto PEG-PtCNPs to afford PEG-PtCNPs-RITC. The PEG-PtCNPs showed strong photothermal conversion ability and could bind to multiple organelles. Additionally, it was found that under mild NIR light irradiation, PEG-PtCNPs impaired the cellular cytoskeleton and induced the overexpression of lamin A/C, leading to the inhibition of tumor metastasis. When the cells were irradiated by the NIR laser at a higher power density, irrecoverable destruction to the nuclear membranes was observed and the nuclear delivery of PEG-PtCNPs in the absence of nucleus-targeting ligands was subsequently realized. Thus, this nanoplatform achieved the damage to the cytoskeleton and nucleus by multiorganelle-targeted PTT and showed exceptional performance in tumor ablation and metastasis restriction. This work provides a new solution for targeting multiple organelles to elevate the efficiency of low-temperature PTT.

#### 2.3.4. Synergistic Therapy

Since numerous preclinic and clinic experiments have disclosed that many monotherapies show low efficiency in defeating tumor metastasis/reoccurrence and some adverse side effects like high toxicity to normal cells and damage to immune system during the past few decades, many researchers have devoted themselves to conquering these obstacles. The rational combination of conventional strategies has been appreciated as an important breakthrough verified by a myriad of studies, which has opened a new chapter in the field of cancer therapy [146].

As is well known, the presence of hypoxic cells in tumors is considered to be one of the main reasons for the reduction of the effectiveness of radiotherapy, certain chemotherapeutic drugs, and phototherapy [147], and the insufficient blood flow within tumor tissues impedes the delivery of various therapeutic agents [111]. Fortunately, the association of mild hyperthermia treatment with other therapeutic modalities has obtained many encouraging results. It was found that at a slightly higher temperature the tumor hypoxic environment can be ameliorated due to the accelerated blood flow [147–149]. Additionally, increased vascular permeability nearby the heated region can be observed, which may contribute to the enhanced permeability and retention (EPR) effect (the key of the passive accumulation of drugs in tumor tissues). Thus, low-temperature PTT, which can induce mild hyperthermia within tumors, may have considerable potential in improving the effects of other therapies. In the meantime, it has been widely recognized that NPs can passively accumulate in tumors due to the EPR effect and actively target tumors through being modified by some molecules or motifs capable of recognizing and interacting with tumors or TME [150]. Moreover, the utility of nanostructures consisting of PTAs and other agents like chemodrugs, radiosensitizers, and photosensitizers can integrate multimodal treatments in one nanoplatform. Therefore, with the assistance of nanotechnology, we can remarkably improve the cancer cell-killing performance of other therapies in combination with low-temperature PTT. On the other hand, other treatment results can sensitize cancer cells to be more vulnerable to mild heat, thus enhancing the tumor eradication effect of low-temperature PTT. Besides, low-temperature PTT possesses other unique advantages when it is combined with different therapeutic modalities, which will be discussed in the following parts.

*(1) Chemotherapy*. Chemotherapy, surgical resection, and radiotherapy (RT) are considered as the three major tactics for cancer treatments. Chemotherapy plays an important role in clinical cancer therapy and a large number of novel cancer therapeutic strategies and agents have been developed up till now [151–154]. Nevertheless, there are several problems that are desperately imperative to be solved, e.g., indiscriminative accumulation and cellular uptake leading to incomplete tumor eradication and unavoidable damage to normal tissues, the development of multidrug resistance (MDR) compromising the therapeutic performance of the chemodrugs, and unsatisfactory therapeutic effects of the drugs used at safe doses [155–157]. Thus, it is of great significance to develop a platform which can efficiently kill tumors at a relative low drug dose.

**Figure 6:**
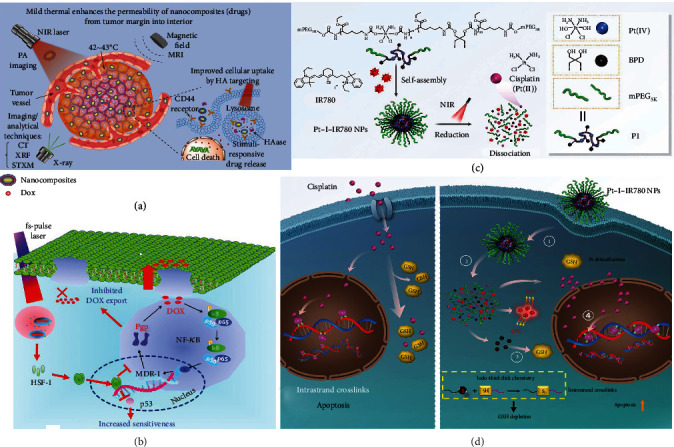
(a) Scheme illustrating the enhancement of tumor-interior permeability accomplished by Gd-hybridized plasmonic Au-nanocomposites in multimodal imaging-guided cancer therapy. Reprinted with permission from Reference [160]. Copyright 2016, Wiley-VCH. (b) Discipline of defeating cancer cell drug resistance under fs-pulse laser irradiation. Reprinted with permission from Reference [171]. Copyright 2014, Wiley-VCH. (c) Scheme showing the construction and NIR light- and GSH-mediated dissociation of Pt-I-IR780 NPs. (d) Scheme showing the significantly elevated cancer treatment efficiency of Pt-I-IR780 NPs (compared with cisplatin) achieved via low-temperature PTT-enhanced chemotherapy. (c, d) Reprinted with permission from Reference [177]. Copyright 2020, American Chemical Society.

To date, an agreement has been reached that combining chemotherapy with PTT can make a practical contribution to improving the anticancer outcomes [158]. First, PTT may achieve spatiotemporally-controlled drug delivery by adjusting the location, power density, and working time of NIR/visible light. Second, hyperthermia can render cancer cells more accessible and vulnerable to chemotherapeutic drugs. Third, the heat generated by PTAs can stimulate the chemodrugs' intratumoral penetration and accumulation due to increased tumor blood flow and vascular permeability. In addition, due to the reduced phototoxicity and enhanced stability of some chemodrugs at moderate temperatures, decreasing the temperature used to kill tumors by controlling light intensity, blocking HSPs, or interfering with other thermoresistance-related signal pathways makes the combined use of chemotherapy and mild PTT an appealing choice for cancer treatments. Furthermore, the administration of chemodrugs can compensate for the insufficient heat damage, thus realizing low-temperature PTT [159–177]. For instance, Sherlock et al. fabricated an ultrasmall nanocrystal (FeCo/GC) containing a single crystalline iron-cobalt core surrounded by a single- or few-layer graphitic carbon (GC) shell, which could serve as a PTA and an MRI contrast agent [159]. Further, doxorubicin (DOX) was loaded on the GC shell by *π*-*π* stacking interaction to form FeCo/GC-DOX. Under acidic conditions in TME and endosomes/lysosomes, the *π*-*π* stacking interaction was weakened, leading to the pH-sensitive release of DOX. Under NIR light irradiation, FeCo/GC-DOX exhibited enhanced cellular uptake, which was determined by MRI, DOX fluorescence measurements, and flow cytometry. The enhanced cellular uptake and the elevated chemotherapeutic efficacy of DOX under mild hyperthermia dramatically enhanced the toxicity of FeCo/GC-DOX toward breast cancer cells. Wang et al. also found that mild hyperthermia could help DOX to penetrate the extravascular space and transiently alter the tumor interstitial permeabilization by using DOX-loaded Gd-hybridized plasmonic Au nanocomposites [160]. The authors also confirmed that after further HA modification, the obtained nanocomposites could target CD44-overexpressing cancer cells and achieve drug release mediated by HAase, further leading to enhanced tumor elimination (Figure 6(a)). Besides, a large number of other PTAs, including hollow CuS NPs [161], Rb_x_WO_3_ nanorods [162], gold nanomaterials [163], black phosphorus nanosheets [164], PDA NPs [165, 166], ICG or polypyrrole- (PPy-) containing nanomaterials [166–169], and hollow mesoporous organosilica nanocapsule [170], have been employed to cooperate with various clinical chemotherapeutic drugs, such as paclitaxel, docetaxel (DTX), and gemcitabine, to realize synergistic low-temperature PTT/chemotherapy.

Drug-resistant cancer cells tend to express a series of membrane transporters represented by the ATP-binding cassette transporter (ABC) family to export diverse cytotoxic drugs, and in the meantime induce the mutation of proapoptosis genes like TP53 to escape from apoptosis, then resulting in the elevation of their endurance to the therapeutic agents. Fortunately, it is reported that the mild heat produced by PTAs may bring us encouraging hope. Wang et al. found that the moderate heat produced by AuNR-based mesoporous silica nanocarriers (Au@SiO_2_) under the 780 nm femtosecond pulsed laser irradiation (3.2 W cm^−2^, 10 min) not only evoked the obvious and long-lasting expression of the heat shock factor-1 (HSF-1) capable of restricting the NF-*κ*B pathway, which is involved in the formation of P-glycoproteins (Pgps), but also elicited the downregulation and decomposition of mutant p53 in resistant cells to render cancer cells susceptible to chemodrugs [171]. The authors also loaded DOX inside the mesoporous shell of Au@SiO_2_, and found that the resultant nanoplatform (termed Au@SiO_2_-DOX) could avoid Pgp-mediated DOX efflux via the cellular internalization and lysosomal accumulation of Au@SiO_2_-DOX. Moreover, under localized laser, Au@SiO_2_-DOX enhanced the cancer cells' sensitiveness to DOX due to the suppressed mutation of p53 (Figure 6(b)). Further, Du et al. employed cocktail chemotherapy, i.e., administration of two or more than two drugs, and low-temperature PTT at the same time to defeat cancer MDR [172]. They synthesized a dual drug-paired polyprodrug NP via the precipitation copolymerization of dopamine-DOX prodrug and subsequent cisplatin coordination. The NPs showed intracellular pH-responsive drug release and photothermal conversion capacity. Under mild hyperthermia, both the reversal of MDR and tumor eradication were achieved by the synergistic treatment.

To further decrease undesirable drug leakage and increase the drug release efficiency in targeted tumors, researchers have introduced some internal and external (as for mild PTT, it is generally NIR light irradiation) stimulus-responsive properties to the drug delivery systems. For example, low pH is a widely used stimulus for specific drug delivery. On the one hand, TME is weakly acidic mainly due to the abnormal metabolism and proton pump overexpression of tumor cells [111]. On the other hand, many NPs undergo the clathrin-dependent endocytosis to enter cells, which leads to the subsequent enzymatic degradation in the endosomes/lysosomes, where the pH is low [178]. In the past few years, some synergistic low-temperature PTT/chemotherapy nanoplatforms combining NIR light irradiation-triggerable and pH-responsive properties showed great capacity in controlling the delivery and release of drugs and achieved satisfactory cancer therapeutic effect [172–176]. For example, Lu et al. prepared copper sulfide-doped periodic mesoporous organosilica nanoparticles (CuS@PMOs) with excellent drug loading capacity and biodegradability [176]. Then, DOX was loaded into CuS@PMOs to obtain DOX-CuS@PMOs. The nanosystem could release DOX molecules upon the triggering of three stimuli—intracellular GSH, acidic environment in TME, and external mild laser irradiation. Besides, it was found that mild heat generated by CuS NPs exposed to the NIR laser could remarkably enhance the cellular uptake of DOX-CuS@PMOs, leading to the enhanced chemotherapeutic efficacy. In addition to the weakly acidic pH of TME, the overexpression of GSH in cancer cells can be also used to achieve stimuli-responsive drug release. Recently, a NP based on the DNA alkylating agent cisplatin was prepared by using an amphiphilic polymer containing a platinum(IV) (Pt(IV)) prodrug and pendant iodides [177]. On the one hand, the Pt(IV) on the main chain of the polymer could be reduced by GSH to obtain cisplatin and result in the breakage of the polymer. On the other hand, iodides on the side chains of the polymer depleted GSH via the iodo-thiol click chemistry, which circumvented the detoxification of cisplatin caused by GSH. Given that cisplatin is usually used in intraperitoneal hyperthermia perfusion therapy in clinical cancer treatment, the authors further introduced a PTA, IR780, into this platform. The obtained Pt-I-IR780 NPs under NIR light irradiation produced mild hyperthermia to promote GSH-mediated reduction of Pt(IV) and iodo-thiol click reaction (Figure 6(c)). Importantly, it was found that mild hyperthermia could facilitate cisplatin to form preferable interstrand crosslinks with DNA rather than intrastrand crosslinks. Since interstrand crosslinks are considerably more harmful to cancer cells than intrastrand crosslinks because of the difficulty of DNA interstrand repair, the Pt-I-IR780 NPs could realize enhanced cisplatin-mediated cancer chemotherapy (Figure 6(d)). This platform not only solves the problem of the unsatisfactory cisplatin toxicity to cancer cells but also achieves low-temperature PTT-enhanced chemotherapy. It is also believed that this work will pave the way for the further clinical translation of the combination of cisplatin-based chemotherapy and hyperthermia.

**Figure 7:**
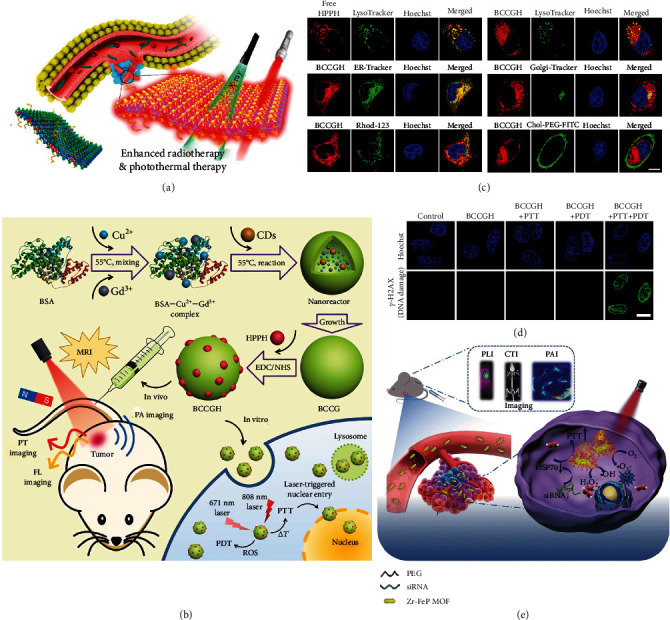
(a) Schematic illustration of the PEG-modified Gd^3+^-doped WS_2_ nanoflake-mediated enhancement of RT and PTT under X-ray and NIR light irradiation. Reprinted with permission from Reference [187]. Copyright 2015, American Chemical Society. (b) Synthesis of BCCGH and its application for multimodal imaging and combined PTT and PDT. (c) Confocal images showing the localization of free HPPH and BCCGH in the different organelles of A549 cells. Hoechst 33342 (Hoechst), ER-Tracker Green (ER-Tracker), rhodamine 123 (Rhod-123), LysoTracker Green (LysoTracker), Golgi-Tracker Green (Golgi-Tracker), and cholesterol-polyethylene glycol (PEG)_2k_-fluorescein isothiocyanate (Chol-PEG-FITC) were adopted to visualize the nucleus, ER, mitochondrion, lysosome, Golgi apparatus, and plasma membrane, respectively. Scale bar: 10 *μ*m. (d) Confocal images of Hoechst- and FITC-labeled *γ*-H2AX-stained A549 cells after various treatments as indicated. Scale bar: 10 *μ*m. (b–d) Reprinted with permission from Reference [197]. Copyright 2018, American Chemical Society. (e) Scheme illustrating the use of siRNA/Zr-FeP MOF nanoshuttles for multimodal imaging and synergistic low-temperature PTT and PDT. Reprinted with permission from Reference [194]. Copyright 2018, Wiley-VCH.

*(2) RT*. Radicals have strong redox activity and can lead to irreversible damage to some vital biomolecules like proteins, DNA/RNA, and lipids [179–181]. RT, which uses ionizing radiation (e.g., X-ray) to directly and/or indirectly react with organic molecules for generating toxic radicals to kill malignant cells, has become a key cancer therapeutic approach to elevate the cancer cells' mortality and decrease unwanted side effects. In addition, employing RT to modulate DNA repair, cell cycle checkpoints, and related signal transduction pathways has also been considered as an encouraging tactic to affect the cellular fates [182]. In the past decades, there have been a large number of studies combining RT and PTT for elevating tumor elimination efficiency [183, 184]. The effect of RT may render cancer cells more sensitive to mild hyperthermia, so the association with RT provides the possibility of realizing low-temperature PTT. Actually, a host of researches have already demonstrated that the synergistic low-temperature PTT/RT has great potential in defeating cancers [185–189]. For example, Chen et al. fabricated an ^131^I-labeled and PEG-coated reduced GO (RGO) (^131^I-RGO-PEG) [186]. Under mild NIR light irradiation, the RGO with strong NIR absorption realized mild hyperthermia. Meanwhile, ^131^I-RGO-PEG exhibited high-energy X-ray emission capacity due to the radionuclide ^131^I, thus killing cancer cells through combined low-temperature PTT/RT. Besides utilizing radionuclides, some nanomaterials capable of converting light into heat and attenuating X-ray have also been exploited to fabricate nanoplatforms that show outstanding tumor eradication performance via synergistic low-temperature PTT/RT. For instance, Cheng et al. synthesized PEG-modified Gd^3+^-doped WS_2_ (a typical type of two-dimensional transition metal dichalcogenides) nanoflakes with strong NIR absorption and X-ray attenuation capacity (Figure 7(a)) [187]. The nanoflakes could also be used as a competent contrast agent for X-ray computed tomography (CT), PA, and MR imaging. Similarly, Yu et al. prepared an all-in-one theranostic platform for synergistic mild PTT/RT and CT imaging [188]. The platform was composed of Bi NPs capped with thiols (via introducing 1-dodecanethiol) capable of protecting the NPs from oxidation and then modified with PEGylated phospholipids. In addition, inspired by the fact that mild hyperthermia is able to improve the tumor oxygenation through increasing the blood flow thus relieving the hypoxia-associated radioresistance, Song et al. constructed a Bi_2_Se_3_ hollow nanocube (HNC) (an NIR PTA) and then loaded GA into the its cavity and decorated the HNC with HA via redox-cleavable disulfide bonds to afford Bi_2_Se_3_ HNC-s-s-HA/GA [189]. The Bi_2_Se_3_ HNC-s-s-HA/GA could target CD44-overexpressing cancer cells, achieve GSH-sensitive GA release, and inhibit HSP90, and could hence accomplish more efficient low-temperature PTT under mild NIR laser irradiation. Moreover, both the X-ray attenuation capability of Bi_2_Se_3_ and the relief of hypoxia of TME due to mild PTT notably enhanced the efficacy of RT. It is noteworthy that under X-ray irradiation, Bi_2_Se_3_ HNCs could realize enhanced RT for eradicating tumor cells without the restriction of depth. Collectively, the above investigations demonstrate that RT represents a robust modality to enhance the efficiency of mild PTT to combat cancer.

*(3) PDT*. PDT is a treatment strategy that makes use of ROS generated by photosensitizers (PSs) exposed to certain light irradiation to cause lethal damage to cancer cells and tissues. Similar to PTT, PDT has an outstanding advantage of spatiotemporally-controlled selectivity and noninvasiveness. Nevertheless, PDT has been confronted with the obstacle that the administration of a high dose of PSs and the use of a strong laser may lead to unexpected phototoxicity to healthy tissues and unsatisfactory cure efficiency. Thanks to the development of nanotechnology, PDT can be facilely combined with PTT by designing desirable nanoplatforms which can potentiate the therapeutic efficacies via a synergistic effect [190–192]. On the one hand, mild heat can remarkably facilitate blood flow and supply sufficient oxygen for potentiating the efficacy of PDT [193, 194]. On the other hand, PDT can elevate the thermal sensitivity of cancer cells [194, 195]. In this way, mild laser irradiation can be adopted in PTT to achieve satisfactory anticancer performance with the assistance of PDT. In the past few years, the synchronous implementation of mild hyperthermia induced by NIR light irradiation and PDT has attracted increasing interest and exhibited remarkable credibility [196–200]. For instance, Liu et al. loaded a photodynamic agent chlorin e6 (Ce6) onto PEGylated MoS_2_ nanosheets (in which MoS_2_ nanosheets served as a PTA) for synergistic PDT/mild PTT [196]. In another example, our group used a protein-templated reaction to fabricate a BSA-stabilized, carbon dot-mediated, and Cu/Gd-doped nanodot (termed BCCG), which could serve as an eminent PTA [197]. We first synthesized CDs through the hydrothermal treatment of *L*-cysteine and *o*-phenylenediamine and then added these CDs into the pre-prepared BSA-Cu^2+^-Gd^3+^ complexes (Figure 7(b)). After covalently conjugating 2-(1-hexyloxyethyl)-2-devinyl pyropheophorbide-*α* (HPPH), a PS, onto the surface of BCCG, the obtained nanoagents (termed BCCGH) showed excellent photothermal/photodynamic and fluorescence (FL)/magnetic properties to achieve FL/PA/MR/photothermal imaging-guided cancer therapy (Figure 7(b)). Due to their ultrasmall size and negatively charged surface, BCCGH had an advantage in enhanced tumor accumulation. Meanwhile, the ultrasmall size endowed the nanoagents with decreased systemic toxicity via their rapid renal excretion and increased therapeutic efficacy via deep tumor penetration. Moreover, the nanoagents could escape from lysosomes/endosomes and were mainly distributed in the large-volume organelle (endoplasmic reticulum) around the nucleus (Figure 7(c)). After mild laser irradiation, BCCGH showed significantly increased cellular uptake and exhibited nucleus-targeting capacity, thus greatly improving the anticancer therapeutic effect of PDT (Figure 7(d)). Finally, the nanoagents were found to be able to significantly eradicate tumors and thoroughly restrain the possible tumor recurrence. This work verifies that the combination of low-temperature PTT and PDT provides a promising strategy for cancer treatments.

Besides, by choosing the PSs that have overlapping absorption spectra with the PTAs and considering other issues such as the construction rationality of the combinational PTA/PS nanoplatforms, several studies successfully accomplished the combination of low-temperature PTT and PDT under a single laser, and in the meantime obtained other favorable characteristics, such as the multimodal imaging capacity [194, 198, 199], the specific nucleolus accumulation feature [198], and intrinsic GSH-responsive biodegradability [199]. For example, Zhang et al. reported a multifunctional zirconium-ferriporphyrin metal-organic framework (Zr-FeP MOF) nanoshuttle which not only acted as a PTA but also served as a catalyst for converting H_2_O_2_ and O_2_ into ·OH and ^1^O_2_ for PDT [194]. After being modified with PEG, the nanoshuttle was further loaded with the HSP70 siRNA, an inhibitor of HSP70, to afford the final product termed siRNA/Zr-FeP MOF for mild hyperthermia (Figure 7(e)). Under NIR light irradiation, this nanoplatform demonstrated excellent tumor growth suppression effect via trimodal (photothermal/PA/CT) imaging-guided synergistic low-temperature PTT/PDT. Likewise, Wu et al. fabricated 17-AAG-encapustaed hollow mesoporous organosilica nanoparticles (HMONs), which were capped with BSA­iridium oxide (IrO_2_) NPs and conjugated with PEG to obtain 17-AAG@HMONs-BSA-IrO_2_-PEG (AHBIP) for mild PTT and PDT [199]. The AHBIP showed the catalytic activity of converting H_2_O_2_ into O_2_ attributed to BSA-IrO_2_ NPs, which endowed this nanoplatform with two advantages—(1) the remission of tumor hypoxia, thus enhancing the efficacy of PDT, and (2) the reduction of the inflammatory cytokines induced by H_2_O_2_, thus alleviating excessive inflammation. In another example, Li et al. chose IR820 (a cyanine dye) to serve as both an NIR PTA and a PS to formulate a novel IR820/DTX-coloaded nanoplatform based on methoxy PEG-PCL (mPEG-PCL) micelle, and positively-charged PCL-grafted poly(ethylene imine) (PCL-*g*-PEI) which could stabilize the negatively charged IR820 via electrostatic interaction was introduced into the micelle [200]. It was found that the NIR absorption of IR820 was weakened due to the presence of PCL-*g*-PEI, which was beneficial for low-temperature PTT. Meanwhile, the ability of IR820 in producing singlet oxygen was scarcely influenced by PCL-*g*-PEI. Thus, this micellar nanosystem could simultaneously achieve mild PTT, efficient PDT, and DTX-mediated chemotherapy. Moreover, after further modification with a homing peptide, Lyp-1 (CGNKRTRGC), the resulting nanosystem displayed excellent antitumor performance. This work illustrates that, IR820, an agent that can simultaneously realize PTT and PDT, provides a choice for constructing novel nanoplatforms for achieving low-temperature PTT-involved synergistic cancer therapy.

**Figure 8:**
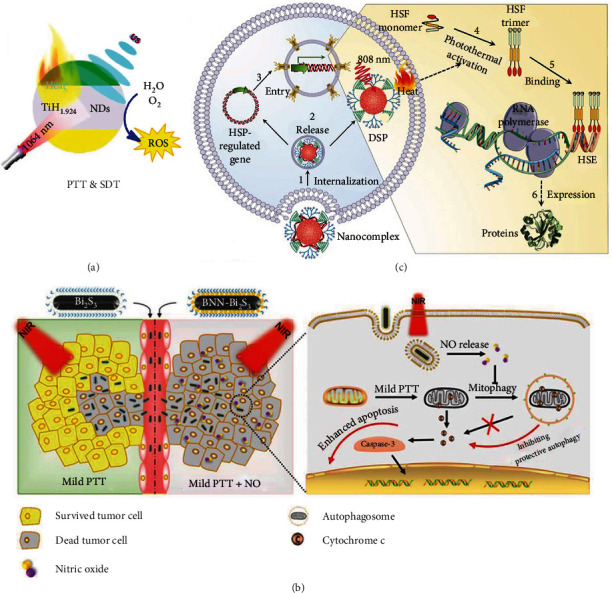
(a) Scheme showing the use of TiH_1.924_ nanodots (NDs) for SDT and PTT. Reprinted with permission from Reference [202]. Copyright 2020, Nature Publishing Group. (b) Scheme describing the use of Bi_2_S_3_ for mild PTT, and the use and working mechanism of BNN-Bi_2_S_3_ for combined NO-mediated gas therapy and mild PTT. Reprinted with permission from Reference [214]. Copyright 2019, Wiley-VCH. (c) Schematic illustration of DSP-mediated gene delivery and the photothermal activation of gene expression under NIR light irradiation. Reprinted with permission from Reference [217]. Copyright 2017, Wiley-VCH.

*(4) Sonodynamic Therapy, Pyroelectric Dynamic Therapy, and CDT*. Apart from the traditional ROS generation methods, a number of strategies have been proposed to achieve ROS-mediated tumor eradication, which may render cancer cells more vulnerable to mild heat, thereby accomplishing low-temperature PTT. For instance, it is noteworthy that sonodynamic therapy (SDT) utilizing materials capable of producing ROS upon ultrasonication has paved a new way for cancer treatment [201]. For instance, Gong et al. found that the synthesis of titanium hydride (TiH_1.924_) nanodots could be easily achieved by liquid-phase exfoliation treatment of TiH_1.924_ powder in the presence of solvents with appropriate surface energy like dimethyl sulfoxide/*N*-methyl pyrrolidone (DMSO/NMP), DMSO, PEG 200, and NMP [202]. They chose NMP to fabricate TiH_1.924_ nanodots because NMP offered outstanding exfoliation efficiency and the resultant nanodots exhibited uniform sizes and morphology. The nanodots served as an outstanding PTA and excellent sonosensitizer, which could achieve the combination of low-temperature PTT and SDT under NIR light irradiation and ultrasonication (Figure 8(a)). Besides SDT, Tang et al. fabricated a unique pyroelectric material, SnSe-polyvinylpyrrolidone (SnSe-PVP) nanorods, which converted the temperature fluctuations during the heating/cooling processes into electrical energy to react with water to obtain ROS [203]. They synthesized the SnSe nanorods by using a simple high-temperature thermal decomposition process and modified the surface of the obtained hydrophobic SnSe nanorods with PVP to enhance the hydrophilicity and biocompatibility. Under mild NIR-II light irradiation, SnSe-PVP not only realized high-performance photothermal conversion for low-temperature PTT but also accomplished pyroelectric dynamic therapy (PEDT). Moreover, in the past few years, CDT which utilizes metal ions (e.g., iron ions) to stimulate Fenton reaction capable of converting H_2_O_2_ into highly toxic ·OH to induce cancer cell death has been considered as an appreciated method to conquer cancers [116, 153, 204, 205]. Considering that elevation of temperature can enhance the Fenton reaction efficiency and ·OH productivity [206, 207], She et al. fabricated red blood cell (RBC) membrane-coated FeS_2_ (FeS_2_@RBCs) with strong absorption at the NIR-II window and Fenton reaction activity to realize the mild PTT-augmented CDT [208]. Similarly, Guo et al. constructed W_18_O_49_ nanorods for PAI-guided combined low-temperature PTT and CDT to treat cancer [209]. The authors utilized a one-step pyrolysis method to synthesize hydrophobic oleyl amine-coated W_18_O_49_ nanorods and then employed 1,2-distearoyl-*sn*-glycero-3-phosphoethanolamine-*N*-[methoxy(polyethylene glycol)-2000] (DSPE-PEG_2000_) to prepare hydrophilic W_18_O_49_ nanorods via hydrophobic self-assembly. The finally obtained W_18_O_49_ nanorods showed both photothermal conversion and Fenton-like reaction capacities with satisfactory PAI contrast. The in vivo experiments demonstrated that the nanorods had outstanding potential for cancer treatment. These examples emphasize the feasibility of employing new ROS-generating cancer therapeutic strategies for achieving improved low-temperature PTT performance.

*(5) Gas Therapy*. Gas therapy utilizing gasotransmitters, which play an indispensable role in regulating diverse physiological behaviors, has been regarded as a “green” option of cancer treatment [210]. As for nitric oxide (NO), a high concentration of NO can directly lead to cell death by modulating various pathways, such as generating oxidative and nitrosative stresses, inhibiting DNA synthesis and repair, suppressing cellular respiration, and stimulating inflammatory reactions [211, 212]. Besides, it has been found that NO also displays advantages in enhancing the efficiency of other therapeutic modalities [213]. First, NO can sensitize the multidrug-resistant cells to chemodrugs. Second, NO is capable of acting as a radiosensitizer to assist RT in treating hypoxic tumors. Third, in the presence of ROS, NO can be oxidized into highly reactive peroxynitrite (ONOO^−^) to strengthen oxidative stress for killing cancer cells. Nevertheless, how to deliver exogenous gasotransmitters safely and precisely has become one of the most imperative questions in the gas therapy field. Hence, some recent state-of-the-art studies have focused on the design of various endogenous and/or exogenous stimulus-responsive NO-releasing nanostructures and their application in on-demand NO-sensitized synergistic cancer therapy [213]. Among them, the NIR light used in mild hyperthermia has been suggested to be an appropriate exogenous stimulus and the combination of low-temperature PTT and NO-mediated gas therapy has shown greatly improved cancer therapeutic effect. For example, Zhang et al. proposed that utilizing NO in mild PTT could improve the mild PTT's capability of eradicating tumors [214]. They used Tween 20 (a commercial surfactant) to modify the surface of bismuth sulfide (Bi_2_S_3_) NPs to improve Bi_2_S_3_ NPs' dispersity in physiological solutions and then added a hydrophobic NO donor, *N*,*N*′-di-sec-butyl-*N*,*N*′-dinitroso-1,4-phenylenediamine (BNN, which is relatively stable below 60°C) to form the final product BNN-Bi_2_S_3_ nanocomposites via hydrophobic interaction. Upon NIR light irradiation, the high local temperature on the surface of Bi_2_S_3_ NPs could cause on-demand NO release, and the surrounding temperature of the nanocomposites was kept at a low level to achieve mild PTT. Moreover, the released NO could suppress cancer cell autophagy-mediated self-repair which was triggered by mild PTT, and promote cancer cell apoptosis, thus rendering tumor vulnerable to mild heat (Figure 8(b)). Meanwhile, NO could diffuse homogeneously in the solid tumor to further enhance the effect of NO-sensitized low-temperature PTT for defeating cancer. Likewise, S-nitrosothiol, a thermosensitive donor of NO, was conjugated onto the core-shell structure of Au@SiO_2_ nanomaterials (serving as PTAs) by You et al. [215]. Then, the authors decorated Au@SiO_2_ with PEG, and loaded PES, an inhibitor of HSP70, into the cavity of the SiO_2_ shell. Upon NIR light illumination, the NO produced by S-nitrosothiol and PES-mediated inhibition of HSP70 could collaboratively induce cell apoptosis or necrosis, thereby strengthening the cancer therapeutic effect of the synergistic low-temperature PTT/gas therapy.

*(6) Gene Therapy*. Given that nucleic acids are the determinant of almost all bioactivities from the molecular to physiological level, gene therapy delivering exogenous nucleic acids with the help of suitable vectors has gradually become one of the most prevalent cancer therapeutic strategies. As mentioned above, the major challenge in this field is designing delivery tools capable of releasing nucleic acids accurately and controllably as well as protecting them from degradation. It is suggested that PTT may provide an ideal way for nucleic acid delivery/release [109], because the heat induced by light can be employed as a trigger of nucleic acid release and can be spatiotemporally controlled. Besides, it has been found that the mild photothermal effect of PTAs can significantly facilitate the intracellular uptake of therapeutic nucleic acids and thus elevate the cancer treatment efficacy of gene therapy [109, 216]. In the past few years, some studies have suggested that the codelivery of PTAs and therapeutic nucleic acids encoded with HSP70 promoters provides the possibility of precisely regulating the expression of therapeutic genes under mild photothermal conditions [217–219]. The moderate increase of temperature caused by PTAs enables the heat shock factor (HSF) to transform from an inactive monomer to an active trimer, which is capable of translocating into the nucleus, and meanwhile, the therapeutic nucleic acids encoded with HSP70 promoters can enter the nucleus. Then, the intranuclear HSF trimer binds to the heat-shock element of the HSP70 promoter and activate the expression of the therapeutic genes cloned downstream of the HSP70 promoter. For example, Lyu et al. designed a dendronized semiconducting polymer (DSP) consisting of three components: the hydrophobic semiconducting backbone, the cationic third-generation polyamidoamine (PAMAM3) side chains, and the PEG segments, serving as the PTA, the gene vector, and the water-solubility enhancer, respectively [217]. After loading HSP-regulatable gene plasmids to DSPs, the authors obtained the nanocomplexes which achieved not only the efficient delivery of genes but also mild heat-controlled gene expression (Figure 8(c)). Similarly, Liu et al. selected PB and therapeutic plasmid DNA consisting of HSP70 promoter, tumor suppressor p53 gene, and green fluorescent protein (GFP) gene (acting as a reporter gene) to achieve thermocontrolled synergistic gene therapy/PTT [218]. It was observed that mild NIR light irradiation (temperature: ~41°C) could stimulate the HSP70 promoter for activating tumor suppressor p53-dependent apoptosis, and meanwhile, strong NIR light irradiation (temperature: ~50°C) resulted in the photothermal ablation of tumors through cellular dysregulation and necrosis. This work emphasizes the feasibility of combining low-temperature PTT-assisted gene therapy and conventional PTT to achieve effective tumor eradication by both apoptosis and necrosis.

Moreover, considering that oncolytic adenovirus (Ad) is widely recognized as a promising candidate for cancer gene therapy, and some studies demonstrate that mild hyperthermia can improve the anticancer effect of oncolytic Ads in vitro [220–222], Jung et al. fabricated an AuNR-mediated mild hyperthermia nanoplatform for enhanced oncolytic Ad gene delivery [223]. They combined the oncolytic Ad expressing VEGF promoter-targeted artificial transcriptional repressor zinc-finger protein with AuNR-mediated mild PTT. Under NIR laser irradiation, the mild heat generated by AuNRs notably promoted the endocytosis of oncolytic Ads, transgene expression, viral replication, and subsequent cytolysis of head and neck cancer cells, thus realizing the elevated efficiency of oncolytic Ads in cancer treatment.

Besides HSPs, Bcl2-associated athanogene domain 3 (BAG3) is also responsible for protecting cells from thermal attacks and developing cellular thermoresistance [224]. Inspired by this fact, Wang et al. used AuNRs to deliver BAG3-targeting siRNA oligomers to silence the expression of BAG3, thus rendering cancer cells vulnerable to low-temperature PTT [225]. The AuNRs were prepared by a seed-mediated growth method with hexadecyltrimethylammonium bromide and were sequentially modified with negatively charged poly(sodium 4-styrenesulfonate) and positively-charged poly(diallyldimethylammonium chloride) to form positive charged nanorods for connecting with BAG3-targeting siRNA oligomers through electrostatic interaction. The experimental results demonstrated that the resultant nanocomplexes exhibited great NIR photothermal conversion ability and the cancer cells were more sensitive to heat with the help of BAG3-targeting siRNA oligomers, thus realizing both gene delivery and low-temperature PTT.

*(7) Immunotherapy*. In the past few decades, immunotherapy has played an unparalleled role in defeating cancers and exhibited satisfactory capability to eradicate both primary and distant tumors [226, 227]. Since the immune system is a critical regulator of the tumor physiology and biology, which may result in the stimulation or suppression of tumor development, growth, invasion, and metastasis, it is important to develop effective immunotherapeutic strategies with the capacity to drive the immune system to pose a lethal threat to tumors rather than help them survive [228–231]. There have been numerous studies which focus on the exploration of cancer immune escape mechanisms and try to elevate the efficiency of immunotherapy [231]. Three major mechanisms have been proposed [231]: (1) the loss of antigenicity which has the potential to induce tumor-specific immune responses, (2) the poor immunogenicity of cancers, which may endow tumors with additional immunosuppressive properties via the overexpression of immune checkpoints and the secretion of suppressive cytokines, etc., and (3) the generation of immunosuppressive TME where the tumor-infiltrating lymphocytes (TILs) like macrophages do not attack cancer cells. In view of these problems, various immunotherapeutic approaches represented by chimeric antigen receptor (CAR) T cell therapy, immune checkpoint blockade (ICB), and cancer vaccines have been proposed and exhibited outstanding treatment performance [232–236]. Nevertheless, due to the heterogeneity and complexity of cancer, immunotherapy may not be able to activate the immune system to prevent tumor reoccurrence and metastasis, and its efficiency may be restrained by the dense extracellular matrix (ECM) and elevated interstitial fluid pressure (IFP) nearby the tumor tissue during the delivery of therapeutic molecules or cells [231, 237, 238]. Fortunately, the collaboration with low-temperature PTT can shed new light on the elevation of the immunotherapeutic efficiency. It has been found that mild hyperthermia can not only directly kill cancer cells but also lead to the partial disruption of ECM, the decrease of IFP, and the increase of the blood perfusion, which is beneficial for the delivery of immunotherapeutic agents and the recruitment and infiltration of lymphocytes. More importantly, mild hyperthermia can produce a favorable TME for immune responses via making dying cancer cells release tumor-associated antigens (TAAs) and damage-associated molecular pattern molecules (DAMPs) to modulate the immune system, consequently promoting the maturation of antigen-presenting cells (APCs), facilitating the secretion of immunostimulatory cytokines, and activating the cytotoxic lymphocytes to defeat tumors [167, 239–242]. In addition, Deng et al. found that the GO-mediated low-temperature PTT could inhibit the M2 polarization of tumor-associated macrophages (TAMs) (M2 phenotype TAMs are beneficial for tumor growth and metastasis) and thus contribute to evoking the immune system [243]. However, the immune responses induced by mild hyperthermia alone are usually not enough to remarkably reverse the tumor-mediated immunosuppressive microenvironment [244]. Therefore, on the one hand, the combination of low-temperature PTT and immunotherapy may pave a promising way for significantly evoking immune systems to eradicate cancers. On the other hand, the synergistic cancer treatment provides the possibility to achieve low-temperature PTT. For example, Chen et al. fabricated poly(lactic-*co*-glycolic acid) (PLGA)-ICG NPs to generate mild heat under NIR light irradiation and found that the mild heat could trigger increased infiltration and accumulation of the chondroitin sulfate proteoglycan-4- (CSPG4-) specific CAR T cells in tumor tissues [242]. This platform shows the eminent performance of mild PTT in elevating the therapeutic efficacy of CAR T cells in solid tumors.

**Figure 9:**
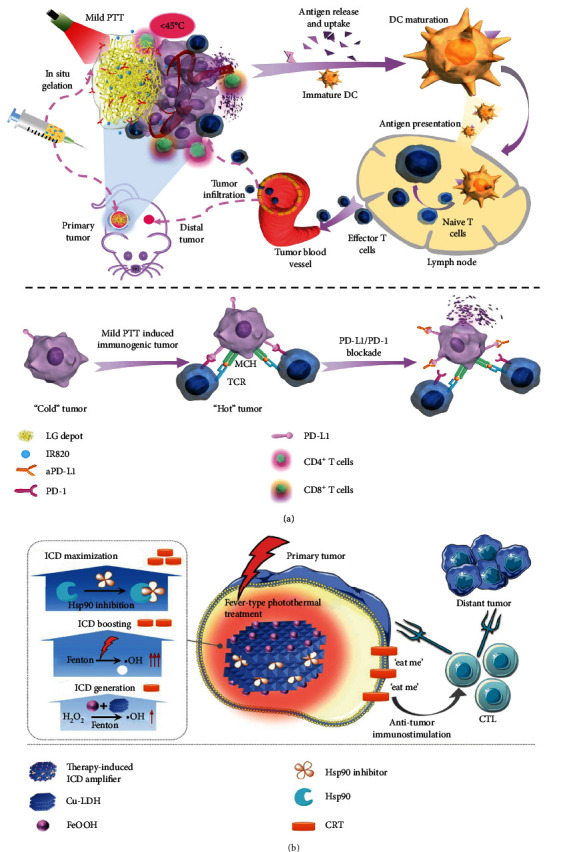
(a) Scheme illustrating the use of the IR820/aPD-L1-encapsulated LG depot for SMPAI. Reprinted with permission from Reference [256]. Copyright 2019, Nature Publishing Group. (b) Scheme depicting the fever-type photothermal treatment and Fenton reaction-enhanced immunotherapeutic effect by using the FeOOH@STA/Cu-LDH nanohybrid. Reprinted with permission from Reference [43]. Copyright 2020, Wiley-VCH.

In addition, ICB with the aim of inhibiting the immune checkpoints to activate immune responses has been widely exploited for the eradication of various cancers. It has been recognized that the two major determinants of the cancer therapeutic effect of ICB-based strategies are the expression of programmed death-ligand 1 (PD-L1) in the tumor tissues and the number of TILs [245–247]. However, a number of studies have demonstrated that some kinds of tumors were not vulnerable to ICB treatments because there are few TILs and low PD-L1 content in these tumor tissues [248–251]. Fortunately, due to its excellent potential for facilitating the recruitment of TILs and its capacity of inducing the elevated expression of PD-L1 on the plasma membranes of cancer cells, mild PTT is able to provide a possibility of remodeling the TME and enhancing the efficacy of ICB-based immunotherapy [239, 251–255]. For instance, Huang et al. encapsulated both IR820 and PD-L1 antibody (aPD-L1), which is capable of interfering with the interaction of programmed death protein 1 (PD-1) with PD-L1, into an injectable lipid gel (LG) depot which could undergo thermally reversible gel-to-sol phase transition [256]. Under the mild heat induced by the NIR light irradiation, the drug-loaded LG depot in sol phase successfully released aPD-L1 for achieving the symbiotic mild photothermal-assisted immunotherapy (SMPAI). This platform shows strong capacity in reversing the immunosuppressive microenvironment via ICB and exhibiting noticeable primary and distal tumor ablation effect (Figure 9(a)). It is noteworthy that the characteristics of this platform such as a consistent retention time in different tumors, the drug exposure dose, and the drug release amount and schedule could be precisely controlled through adjusting the component proportion of LG, changing drug loading amount, and manipulating NIR irradiation, which endowed the platform with great potential for personalized cancer therapy. Recently, Guo et al. designed a “one-for-all” nanosystem based on PEGylated PEI-coated monoelemental bismuthene loaded with siRNA against PD-L1 for mild photothermal immunotherapy [257]. This nanosystem could detach PEG in acidic TME, which resulted in the decreased size and formation of positively charged surface (due to the exposure of PEI) of these NPs, thus enhancing cancer cell uptake. After the endocytosis of the NPs, PEI assisted the NPs to escape from endosomes via the “proton sponge” effect, and meanwhile, the bismuthene-mediated mild hyperthermia could also promote the endosomal escape and induce the controllable release of siRNA against PD-L1. Then, under NIR light irradiation, the bismuthene released to the cytoplasm achieved low-temperature PTT, and it was found that mild hyperthermia stimulated a systemic immune response, recruited abundant TILs, and upregulated the expression of PD-L1 on tumor cells. The above two examples highlight the synergistic effect of low-temperature PTT and ICB-based immunotherapy and provide implications for the design of well-integrated nanoplatforms for cancer therapy.

In addition, cancer vaccines are designed to elicit long-lasting immune activation [258]. However, some studies have demonstrated that most cancer vaccines composed of defined peptide/protein antigens rather than all antigens which are obtained from whole tumor cells have the inferior ability of inducing immunological memory cells to develop an anticancer immunologic memory [259, 260]. Additionally, it is very difficult to collect the whole tumor to prepare a standardized vaccine line consisting of a variety of antigens from whole cancer cells. More importantly, TAAs may vary among tumors and individuals, which further restrains the development of cancer vaccines [261]. With regard to these problems, Chen et al. chose an immunomodulator resiquimod (R848) rather than antigens to develop a low-temperature PTT-mediated cancer vaccine [262]. R848 is the agonist of Toll-like receptors 7 and 8 (TLR-7/8) which are abundant in the immune cells that infiltrate TME, and it has been proved that the stimulation of TLR-7/8 by R848 can activate APCs via facilitating the maturation of DCs and subsequently transforming DCs' phenotype from immunosuppressive to immunogenic [263–265]. Then, with the assistance of diverse proinflammatory cytokines (e.g., interleukin 6 and tumor necrosis factor-*α*) secreted by matured DCs, the antitumor immune responses can be activated. The authors used an amphiphilic copolymer consisting of hydrophobic polyaniline (PANI) and the hydrophilic glycol-chitosan (GCS) backbone to deliver R848 via loading R848 in the hydrophobic core [262]. PANI served as an NIR PTA and achieved low-temperature PTT, and mild hyperthermia showed satisfactory performance in rendering the primary tumor residues more sensitive to immunotherapy. Meanwhile, this platform successfully realized the development of robust and long-term immunological memory against cancer and the effective inhibition of tumor reoccurrence and metastasis. This work verifies that mild PTT can potentiate the immunotherapeutic efficiency of cancer vaccines.

Moreover, immunogenic cell death (ICD), a cell death type that can release DAMPs to activate the immune system to fight against tumors and establish long-term immunosurveillance, is gaining increasing research interest in cancer therapy [266]. It has been found that chemotherapy, PTT, RT, and PDT are all able to induce ICD and then stimulate the tumor-specific immune responses [267–269]. During ICD, calreticulin (a significant marker of ICD) can migrate from the endoplasmic reticulum of tumor cells to the plasma membrane and induce dendritic cells to engulf dying tumor cells and their debris. However, the undesirable toxicity to immune cells in harsh treatment conditions may severely affect the process of ICD and the reduced toxicity to cancer cells in mild treatment conditions can decrease the tumor therapeutic efficacy [270, 271]. Meanwhile, overheating can destroy antigens from dying tumor cells leading to the decreased tumor-killing immune responses [272]. Thus, the development of efficacious cancer therapeutic strategies with relatively mild treatment conditions is highly needed. Recently, the combination of low-temperature PTT and ICD-mediated immunotherapy has demonstrated its remarkable capacity of elevating the efficacy of cancer treatments [43, 272, 273]. For example, a triple therapeutic protocol combining low X-ray dose RT, low-temperature PTT, and immunotherapy was developed based on semiconductor heterojunction structured WO_2.9_-WSe_2_-PEG NPs [272]. Under X-ray irradiation, the NPs could catalyze H_2_O_2_ existing in TME to generate nonoxygen-dependent ROS which not only damaged cancer cells but also further triggered ICD. Besides, the NPs exhibited excellent NIR photothermal conversion capacity and it has been found that mild PTT was capable of triggering ICD to some extent. With the administration of aPD-L1, this platform effectively achieved tumor eradication and metastasis inhibition under mild temperature (induced by WO_2.9_-WSe_2_-PEG NPs upon mild NIR laser irradiation (0.5 W cm^−2^)) and low X-ray radiation dose. This work further verifies the ICD-inducing ability of RT and mild PTT and provides a valuable strategy for defeating cancer in a much safer condition. Additionally, Li et al. found that fever-type (i.e., low-temperature) PTT could facilitate the ROS-induced ICD to enhance immunotherapy [43]. They prepared an NIR PTA composed of Cu-containing layered double hydroxide (Cu-LDH), anchored highly reactive FeOOH nanodots which can generate ROS via Fenton reaction onto Cu-LDH, and inserted STA-9090 (an HSP90 inhibitor) into the gaps between the layers of Cu-LDH to promote cancer cell apoptosis at low temperatures, finally obtaining the FeOOH@STA/Cu-LDH nanohybrid. Under NIR light irradiation, the Fenton reaction-induced ICD was sequentially enhanced by mild hyperthermia and HSP90 inhibition and the primary and distant tumors were effectively eliminated by this synergistic therapeutic platform (Figure 9(b)). This work proposes a novel method for strengthening ICD-mediated immunotherapy via the synergistic Fenton reaction/low-temperature PTT. Moreover, the employment of an ICD inducer with mild PTT also shows eminent performance in eradicating tumors. Zhou et al. prepared a porous cobalt sulfide nanomaterial (PCS) via a simple DNA-templated hydrothermal method for PTT and the delivery of EGCG, an HSP70 inhibitor, and oxaliplatin, an ICD inducer [273]. The authors utilized the EGCG-mediated low-temperature PTT as the “first-hit” to kill tumors and the oxaliplatin-mediated ICD as the “second-hit” to eliminate the potential threat of tumor reoccurrence. Moreover, the release of EGCG and oxaliplatin was induced by pH- and heat-trigger, respectively, which realized the controllable drug delivery sequence. This work may inspire more researchers to employ low-temperature PTT to activate the immune system and amplify the cancer elimination effect of immunotherapy.

## 3. Conclusions and Perspectives

In the past few years, low-temperature PTT based on nanomaterials has attracted considerable attention and exhibited promising potential in conquering some important problems in the biomedical region. It has been demonstrated that, with the assistance of nanotechnology, mild hyperthermia induced by NIR laser shows many advantages in elevating the therapeutic efficacy in diverse applications, because the well-designed nanoplatforms containing PTAs can (1) passively penetrate biofilms due to their nanoscale sizes and physicochemical properties or accumulate in the tumor tissues via EPR effect and specifically target bacteria or cancer cells via some integrated bacterium/tumor-targeting molecules or motifs, (2) possess some beneficial characteristics including stimulus-responsive property, organelle-targeting capability, and multimodal imaging capacity, (3) be combined with other therapeutic modalities to achieve synergistic therapies, and (4), as for cancer treatments, achieve significantly increased cellular uptake and tumor accumulation of the administrated therapeutics with the help of mild heat to potentiate the tumor therapeutic effect.

In this review, we have summarized the recent progress of nanomaterial-mediated low-temperature PTT from three aspects—(1) bacterial elimination, (2) wound treatment, and (3) cancer treatment. As for the antibiosis, there are two major obstacles impeding us to develop more effective strategies to avoid bacterial infection. On the one hand, due to the inappropriate utilization of diverse antibiotics, an increasing number of bacteria have become antibiotic-resistant and the antibacterial efficiency of antibiotics has been decreased dramatically. On the other hand, the troublesome biofilms largely hinder the interaction of antibacterial drugs and/or nanoagents with bacteria. Fortunately, it has been noted that mild PTT may render bacteria more susceptible to other treatments, and some novel methods based on low-temperature PTT have been proposed to solve these problems, including the synergistic NO-enhanced PDT/low-temperature PTT and QCS-MoS_2_ nanoflake-mediated mild PTT for realizing bacterial resensitization to antibiotics [59, 60] as illustrated above. In regard to wound healing, it is inspiring that the combination of low-temperature PTT and other wound therapeutic strategies can achieve rapid and long-term wound healing [65–68]. It has been shown that, in the presence of some tissue regeneration-stimulating substances like bioactive ions, mild PTT can not only defeat bacterial infection but also alleviate excessive inflammation and facilitate the angiogenesis, which are crucial for wound healing [65, 66]. Besides, the introduction of “hot spring” effect also provides an encouraging method for treating chronic wounds [67]. To be noted, as being combined with MSC-based therapy, mild hyperthermia mediated by CuS@BSA NPs can enhance the differentiation of MSCs to form fibroblasts for promoting wound healing [68]. In view of cancer therapy, we first summarized the up-to-date strategies for realizing low-temperature PTT for tumor eradication. First, given that HSPs are the major determinant of the generation of thermal resistance and they play an important role in helping cancer cells escape from apoptosis-mediated death, the inhibition of HSPs using small-molecule HSP inhibitors (e.g., GA, Qu, 17-AAG, VER-155008, PES, EGCG, and BIIB021) and siRNAs can not only render tumors more vulnerable to mild heat for achieving low-temperature PTT but also promote the apoptosis-mediated cell death, which may further enhance the cancer treatment effect of mild PTT. Moreover, because both the synthesis of HSPs and the realization of HSP biological functions need energy, cancer starvation therapy which blocks/reduces the nutrient supply and/or exhausts the nutrients can lead to the decreased production of energy, thus inhibiting HSPs to accomplish low-temperature PTT. Second, the combination with autophagy-mediated cancer therapy sheds new light on the therapeutic effect enhancement of mild PTT. It has been found that the regulation of autophagy can contribute to low-temperature PTT for more effective tumor eradication. Third, the combination of organelle-targeting strategy with low-temperature PTT has also been exploited to achieve satisfactory cancer cell-killing effect because many organelles are vulnerable to mild hyperthermia and each organelle plays an indispensable role in maintaining intracellular homeostasis. Fourth, we concluded the synergistic therapy strategies that combine low-temperature PTT with other treatment modalities (e.g., chemotherapy, RT, gene therapy, and immune therapy). On the one hand, mild hyperthermia can markedly promote the tumor accumulation and retention of NPs/drugs via increasing blood flow and changing the compact tumor ECM into a loose one, thus elevating the intratumoral delivery of chemodrugs, radiosensitizers, nucleic acids, immunotherapeutic agents, etc. to achieve better cancer therapeutic outcomes. On the other hand, other treatments can render cancer cells more susceptible to mild hyperthermia, thus realizing low-temperature PTT. Additionally, it has also been verified that mild heat can alleviate the hypoxia of tumor tissues, which paves a promising way for potentiating the effect of oxygen-dependent treatments such as PDT and SDT. Furthermore, mild hyperthermia has the potential for modulating immune systems, i.e., activating the immunosuppressive microenvironment to form the immunostimulatory one [167, 239–243]. Therefore, the combination of low-temperature PTT and immunotherapy can significantly induce the immune responses to attack cancer cells and inhibit the metastasis and reoccurrence of tumors. More importantly, it is noteworthy that nanotechnology plays an unparalleled role in achieving synergistic therapy. By utilizing different synthesis and modification methods, various materials and agents possessing diverse therapeutic effects can be integrated into the nanoplatforms. Besides, the agents in nanosystems usually exhibit increased therapeutic effects and decreased side effects due to the strong drug-loading capacity, exogenous and/or endogenous stimulus-responsive property, and tumor targeting/accumulation ability of the nanocarriers.

Despite the above strategies developed, there are some problems which we must solve. First, although a large number of nanocarriers for low-temperature PTT have been developed and exhibited satisfactory characteristics like the increased drug encapsulation/loading capacity, decreased toxicity to normal cells, and good biocompatibility and biosafety, we still need to develop nanocarriers with stronger drug delivery capability, more desirable biodegradability, and enhanced stability in long-term blood circulation. Second, more strategies that can achieve low-temperature PTT need to be developed. For example, we can design more nanoplatforms for targeting other organelles except the nucleus, mitochondrion, and cytoskeleton and compare the effectiveness of the different strategies to optimize the organelle-targeted low-temperature PTT. Third, there are some novel and promising therapeutic modalities, including ferroptosis, ion-interference therapy, and electrodynamic therapy, which can also be adopted to be combined with low-temperature PTT for realizing satisfactory synergistic therapies. More importantly, although there have been studies focusing on the working mechanisms of the combination of low-temperature PTT and other treatments, we are supposed to figure out the more detailed action mechanisms of these synergistic therapies to propose more effective combinational strategies and more appropriate administration sequences. Likewise, to elevate the effectiveness of using mild PTT to treat bacterial infections and wounds, researchers are suggested to pay more attention to understanding and utilizing the complex mechanisms from various aspects like the behaviors and roles of bacteria or other indispensable types of cells in bacterial invasion and wound development. Moreover, there are few reports about using low-temperature PTT for treating other diseases like neurological disorders, metabolic diseases, and cardiovascular diseases. Therefore, it is suggested that mild PTT can be applied in more biomedical fields in the future.

To summarize, low-temperature PTT is a robust strategy for eliminating bacteria and cancers. We sincerely hope that this review may inspire more researchers to devote their efforts to this fascinating field and achieve significant advances in mild PTT-involved preclinical and clinical researches.

## Figures and Tables

**Table 1:** Strategies for low-temperature PTT for treating bacterial infections and promoting wound healing.

Applications	Strategies	Agents for strategies	PTAs	Reference
Antibiosis	Antimicrobial peptides	MagI	PDA	[58]
	NO-enhanced PDT	L-Arg and ICG	PDA	[59]
	Antibiotic therapy	Ofloxacin	QCS-MoS_2_ nanoflakes	[60]
		Aminoglycoside antibiotics	Red phosphorus NPs	[61]

Wound healing	Bioactive ion-mediated treatment	Hap nanorods	PDA	[65]
		Hap	GO/NCD/Hap films	[66]
		FA	FA	[67]
	MSC-based therapy	MSCs	CuS@BSA NPs	[68]

**Table 2:** Strategies for low-temperature PTT for cancer treatments.

Strategies	Agents for strategies			PTAs	Reference
HSP downregulation	HSP inhibition	Small-molecule HSP inhibitors	GA	dc-IR825	[27]
				ICG	[93]
				Bi@ZIF-8 NPs	[94]
				HMCSs	[95]
				Semiconducting polymer	[96]
				BPQDs	[97]
				GO	[98]
			Qu	Qu-Fe^II^Ps	[99]
				AuNRs	[100]
			17-AAG	Cypate	[101]
			VER-155008	AuNRs	[102]
			PES	PEDOT	[103]
			EGCG	Lu:Nd@NiS_2_ NPs	[104]
			BIIB021	IR780	[105]
		siRNA HSP70 inhibitors		Cypate	[106]
				Gold nanoshells	[107]
				PDA	[108]
	Cancer starvation therapy	DC		GNRs	[113]
		GOx		ICG	[32]
				ICG	[117]
				PB NPs	[118]
		M-NS		M-NS	[119]
		siPKM2		ICG	[120]

Autophagy-mediated cancer therapy	Autophagy inhibition	CQ or chloroquine diphosphate		PDA	[129]
				PDA	[130]
				PDA	[131]
				PDA	[132]
	Autophagy augment	Beclin 1-derived peptide		PDA	[133]

Organelle-targeting strategies	Nucleus	TAT peptides		Pd nanosheets	[14]
				Vanadium carbide QDs	[140]
		Chitosan-coated ruthenium(IV) oxide NPs		Chitosan-coated ruthenium(IV) oxide NPs	[141]
		Hf-HI-4COOH-based NCPs		Hf-HI-4COOH	[142]
	Mitochondrion	Lipophilic iridium(III)		Fe_3_O_4_ NPs	[143]
	Integrin and cytoskeleton	RGD peptides		AuNRs	[144]
	Multiple organelles	PEG-PtCNPs		PtCNPs	[145]

**Table 3:** Low-temperature PTT-involving synergistic therapies.

Therapeutic modality in combination with PTT	Agents (except PTAs)		PTAs	Reference
Chemotherapy	DOX	PEG	GC shell	[159]
		HA	Gd-hybridized plasmonic Au-nanocomposites	[160]
		PVP	Rb_x_WO_3_ nanorods	[162]
		Low-temperature-sensitive liposomes	Gold nanoantennas	[163]
		PEG	PDA and ICG	[166]
		HSA	ICG	[167]
		Poly(acrylic acid-*b*-*N*-isopropylamide-*b*-acrylic acid)	PPy	[169]
		Fe^3+^ carboxylate MOFs		[173]
		—	Au@SiO_2_	[171]
		Meso-2,3-dimercaptosuccinic acid	Fe_3_O_4_	[174]
		Hydroxypropyl-*β*-cyclodextrin (HP-*β*-CD)	Fe_3_O_4_/carbon NPs	[175]
		Periodic mesoporous organosilica NPs	CuS	[176]
	Camptothecin		Hollow CuS NPs	[161]
	Paclitaxel and HSA		Black phosphorus nanosheets	[164]
	SN38 and alendronate		PDA	[165]
	DTX, PLGA, and PEG		PPy	[168]
	Gemcitabine, 17-AAG, hollow mesoporous organosilica nanocapsule, and PEG		ICG	[170]
	DOX and cisplatin		PDA	[172]
	Cisplatin and amphiphilic polymer containing Pt(IV) prodrugs and pendant iodides		IR780	[177]

RT	^131^I and PEG		RGO	[186]
	PEG		Gd^3+^-doped WS_2_ nanoflakes	[187]
	PEG		Bi NPs	[188]
	HA and GA		Bi_2_Se_3_ HNCs	[189]

PDT	PEG and HSP70 siRNA		Zr-FeP MOF nanoshuttles	[194]
	Ce6 and PEG		MoS_2_ nanosheets	[196]
	HPPH		BCCG nanodots	[197]
	Ce6		Amino-rich red emissive CDs	[198]
	HMONs, BSA, PEG, and 17-AAG		IrO_2_	[199]
	DTX, mPEG-PCL, PCL-*g*-PEI, and Lyp-1		IR820	[200]

SDT	PVP		TiH_1.924_ nanodots	[202]
PEDT	PVP		SnSe	[203]

CDT	PEG		FeS_2_@RBCs	[208]
	—		W_18_O_49_ nanorods	[209]

Gas therapy	BNN and Tween-20		Bi_2_S_3_ NPs	[214]
	S-nitrosothiol, PES, and PEG		Au@SiO_2_	[215]

Gene therapy	HSP-regulated gene plasmids		Semiconducting backbones	[217]
	Therapeutic plasmid DNA and PEI		PB	[218]
	HSP-nuclease protein 9 (Cas9) plasmids		Cationic polymer-coated Au nanorods	[219]
	Oncolytic Ads		AuNRs	[223]
	BAG3-targeting siRNA oligomers		AuNRs	[225]

Immunotherapy	CSPG4-specific CAR T cells		ICG	[242]
	aPD-L1, folic acid, and SNX-2112		GO	[255]
	aPD-L1 and LG depot		IR820	[256]
	siRNA against PD-L1, mPEG, and PEI		Bismuthene	[257]
	R848 and GCS		PANI	[262]
	aPD-L1		WO_2.9_-WSe_2_-PEG NPs	[272]
	FeOOH nanodots and STA-9090		Cu-LDH	[43]
	Oxaliplatin and EGCG		PCS nanomaterials	[273]
